# Snow cover mapping with Meteosat third generation FCI: initial evaluation of the European Organisation for the Exploitation of Meteorological Satellites H SAF H43 snow mask product

**DOI:** 10.7717/peerj.20495

**Published:** 2026-01-13

**Authors:** Semih Kuter, Cagri Hasan Karaman, Mustafa Berkay Akpinar, Zuhal Akyurek

**Affiliations:** 1 Department of Forest Engineering/Faculty of Forestry, Cankiri Karatekin University, Cankiri, Turkey; 2 Middle East Technical University Technopolis, HidroSAF Ltd., Ankara, Turkey; 3 Department of Civil Engineering/Faculty of Engineering, Middle East Technical University, Ankara, Turkey; 4 Graduate School of Natural and Applied Sciences/Department of Geodetic and Geographic Information Technologies, Middle East Technical University, Ankara, Turkey

**Keywords:** Remote sensing of cryosphere, Snow cover mapping, Flexible combined imager, VIS/NIR radiometry

## Abstract

This study presents the results of the initial validation of the EUMETSAT H SAF H43 snow cover product, the first operational snow product derived from the Flexible Combined Imager (FCI) onboard Meteosat Third Generation (MTG). The evaluation, covering the period from December 2024 to February 2025, includes a direct comparison with the earlier H34 product, generated from SEVIRI observations onboard Meteosat Second Generation (MSG). Analyses were conducted over three mountainous regions—the Alps, Turkey, and Georgia—using MODIS-based reference snow cover maps and *in-situ* snow-depth measurements from WMO synoptic stations. As snow cover in mountainous terrain plays a critical role in hydrology and water resources management, special attention was given to high-elevation zones (above 1,000 m), where snow is seasonally persistent and detection accuracy most relevant. The evaluation employed standard accuracy metrics—Probability of Detection (POD), False Alarm Ratio (FAR), and Overall Accuracy (ACC)—to assess performance across elevation, aspect, and land-cover classes. Results indicate that H43 provides consistent yet moderate improvements over H34, with miss-rate reductions of up to 40% above 2,000 m elevation and FAR values remaining below 30% across land-cover types. These improvements are supported by the enhanced radiometric performance and 10-min temporal resolution of the FCI sensor, which enable more effective cloud detection and frequent scene refresh. Combined with its near-real-time availability, these features make H43 a promising tool for operational snow monitoring, hydrological forecasting, and early-warning applications across topographically complex regions.

## Introduction

Monitoring and mapping snow cover on a global scale are essential for understanding its role in the Earth’s energy balance and climate system (Hall, Riggs & Salomonson, 1995). Snow’s high albedo allows it to reflect a significant portion of solar radiation back into space, which plays a major role in influencing surface heating and cooling patterns (Hall & Martinec, 1985). Of all land surfaces, snow has the most significant effect on the surface energy balance (Chen, Yang & Yin, 2021). Changes in snow cover, whether in extent or duration, directly influence the Earth’s energy balance, contribute to shifts in climate and generating feedback loops (Pulliainen et al., 2020; Takala et al., 2011).

Beyond its direct impact on energy balance, snow cover also affects numerous Earth system processes. These include atmospheric circulation patterns (Henderson et al., 2018), permafrost dynamics (Philipp et al., 2021), glacier mass balance (Yang et al., 2023; Yu et al., 2023), river discharge (Zhang et al., 2020), snowmelt-driven flooding (Niu et al., 2025), groundwater replenishment (Li & Ma, 2024), and hydropower production (Stucchi et al., 2019). The ongoing changes in snow cover, driven by climate variability, disrupt these processes and alter the balance of the Earth’s systems (Brown & Mote, 2009). Because of its vital role, snow cover is recognized as an essential climate variable by the Global Climate Observing System (GCOS, 2006; Hüsler et al., 2012), and continuous monitoring is crucial for understanding its trends and implications.

Field-based snow measurements, such as manual snow depth (SD) and snow water equivalent (SWE) observations, provide the only direct data on snow cover. However, these measurements are limited by various factors, including errors caused by wind redistribution, sublimation, and the dependence on local terrain and snow accumulation patterns. Additionally, their spatial distribution is often too sparse, particularly in remote, mountainous, and polar regions, making it difficult to capture the full variability of snow cover (Dong, 2018). Ground-based remote sensing methods, such as weather radars and lidar systems, offer broader coverage than *in-situ* measurements, but provide indirect observations of snow, often affected by uncertainties in signal interpretation due to factors like attenuation, beam blockage, and surface clutter (Lundberg et al., 2016). Because neither approach is sufficient for large-scale snow monitoring, satellite-based remote sensing has become the most effective method for obtaining global snow cover estimates (Frei et al., 2012; Ilčev, 2019; Levizzani, Laviola & Cattani, 2011).

Satellite-based snow cover monitoring relies on the interaction between snow and electromagnetic radiation, primarily through two observation methods: (1) optical and thermal infrared (VIS/IR) and (2) microwave and radar (MW) sensing (Dozier, 1989; Nolin, 2010). These complementary techniques provide the foundation for deriving two essential snow parameters on a global scale—snow cover extent (SCE) and SWE.

Optical and thermal infrared sensors detect snow using its strong reflectance in the visible spectrum and low reflectance and emissivity in the shortwave and thermal infrared regions. These characteristics enable accurate identification of snow-covered surfaces under clear-sky conditions. High- and moderate-resolution sensors such as Landsat, Sentinel-2, Moderate Resolution Imaging Spectroradiometer (MODIS), Visible Infrared Imaging Radiometer Suite (VIIRS), and Advanced Very High-Resolution Radiometer (AVHRR) have been widely used to map snow distribution and monitor its seasonal dynamics (Gascoin et al., 2019; Rößler & Dietz, 2022; Stillinger et al., 2023; Zhao et al., 2024).

Landsat and Sentinel-2, offering spatial resolutions of 10–30 m, are particularly effective in mountainous regions, providing detailed mapping and validation data for coarser global products. MODIS and VIIRS, with moderate resolutions (250–1,000 m) and daily global coverage, have been central to long-term operational monitoring of SCE and fractional snow-covered area (fSCA). Optical and thermal infrared techniques, however, are constrained by cloud contamination, low illumination, and terrain shadowing, which can limit data availability during winter or over rugged terrain.

Microwave and radar systems, encompassing both passive radiometers (*e.g*., SSM/I, AMSR-E, AMSR2) and active radar sensors such as Sentinel-1 SAR, can operate independently of daylight and atmospheric conditions. They provide essential information on SWE, snow wetness, and layering properties, as their longer wavelengths can penetrate clouds and, to some extent, vegetation. Passive microwave radiometers support large-scale and continuous SWE estimation but at coarse spatial resolutions (typically 10–25 km) and are sensitive to forest cover, terrain heterogeneity, and variations in snow microstructure (Pulliainen & Hallikainen, 2001; Saberi et al., 2020). Active radar systems offer finer spatial detail and can detect temporal changes in snowpack characteristics; however, their backscatter signal depends on snow density, grain size, and surface roughness, which complicates retrieval in heterogeneous environments.

Despite these limitations, optical and microwave techniques are complementary and advanced VIS/IR and MW snow cover products have been instrumental in the retrieval of these parameters and tracking seasonal and long-term snow variations, contributing to our understanding of snow’s role in climate and hydrology at both regional and global scales (Awasthi & Varade, 2021; Hidalgo-Hidalgo et al., 2024; Metsämäki et al., 2015). Within this framework, geostationary optical sensors such as the Spinning Enhanced Visible and Infrared Imager (SEVIRI) aboard Meteosat Second Generation (MSG) have played a key role in operational, near-real-time snow monitoring. The Flexible Combined Imager (FCI) onboard Meteosat Third Generation (MTG) further advances these capabilities with improved spatial, spectral, and temporal resolution, enabling more accurate snow detection under dynamically changing atmospheric and illumination conditions.

### A brief survey on satellite-derived long-term global snow cover products

Satellite-based optical sensors have been instrumental in monitoring global snow cover over extended periods, providing consistent and objective measurements across diverse landscapes. Operating in the visible and infrared (VIS/IR) spectra, these sensors detect snow through its high reflectance in the visible wavelengths and low reflectance in the shortwave infrared region. Because of the large spatial extent and rapid temporal variability of snow, most operational datasets rely on coarse- to moderate-resolution imagery with daily global coverage.

Key optical satellite systems such as the AVHRR, the MODIS, and the VIIRS have played a central role in building long-term global snow cover records. MODIS, with its daily coverage and moderate resolution, has been the most widely used for global snow mapping since 2000. AVHRR, one of the earliest operational sensors, provided the first continuous snow cover observations dating back to the late 1970s (Akyürek & Şorman, 2002; Hüsler et al., 2012), while VIIRS now extends this legacy with improved spatial, spectral, and radiometric performance (Riggs & Hall, 2020; Stillinger et al., 2023). Products derived from these sensors—including the MODIS snow cover datasets (Hall et al., 2002) and the NOAA Climate Data Record (CDR) of SCE (Estilow, Young & Robinson, 2015)—have significantly advanced climate, hydrological, and environmental research.

While these polar-orbiting sensors provide extensive historical and spatial coverage, their daily revisit time and cloud dependency limit their effectiveness for continuous operational monitoring. To address these limitations, geostationary optical missions such as MSG-SEVIRI instrument and the new MTG-FCI offer much higher temporal resolution—15 min and 10 min, respectively. The merging of multiple consecutive images within a single day can substantially reduce cloud-affected areas; for example, combining 32 SEVIRI observations per day enables roughly a 37% reduction in cloud coverage compared with the MODIS daily product, thereby improving the mapping of regional snow cover extent, especially over mountainous terrain (Sürer & Akyürek, 2012; Sürer, Parajka & Akyürek, 2014). These advances make geostationary sensors a promising complement to polar-orbiting missions for near-real-time and operational snow monitoring. A summary of the main long-term global optical snow cover products is provided in Table 1. Although the Interactive Multisensor Snow and Ice Mapping System (IMS) (https://nsidc.org/data/g02156/versions/1) provides a valuable long-term global record by combining optical, microwave, and *in-situ* observations, it was not included in Table 1 because it is not a purely satellite-derived optical product.

**Table 1:** Summary of satellite-derived global optical snow cover products.

Sensor name	Product name (Abbreviation)	Spatial/Temporal resolution	Coverage area	Availability period	Web page	Timeliness
MODIS (Terra/Aqua)	MOD10A1/MYD10A1	500 m/Daily	Global (Land)	2000–Present	https://nsidc.org/data/MOD10A1	1–2 days
MODIS (Terra/Aqua)	MOD10A2/MYD10A2	500 m/8-Day	Global (Land)	2000–Present	https://nsidc.org/data/MOD10A2	2–3 days
MODIS (Terra/Aqua)	MOD10C1/MYD10C1	0.05° (~5 km)/Daily	Global (Land)	2000–Present	https://nsidc.org/data/MOD10C1	2–3 days
MODIS (Terra/Aqua)	MODSCAG	500 m/Daily	Global (varies with tiles)	2000–Present	https://snow.ucsb.edu	Several days to weeks (research use)
MODIS (Terra/Aqua)	MOD10A1F/MYD10A1F	500 m/Daily (Cloud Gap Filled)	Global (Land)	2000–Present	https://nsidc.org/data/MOD10A1F	2–3 days
AVHRR	AVHRR Weekly Snow Cover Product	4 km/Weekly	Northern Hemisphere	1981–Present	https://www.ncei.noaa.gov	3–5 days
AVHRR	AVHRR CDR Snow Cover Product	0.05° (~5 km)/Daily & Weekly	Global	1981–Present	https://www.ncei.noaa.gov	Several weeks (reanalysis)
AVHRR	Polar Pathfinder Snow Product	1–5 km/Daily	Polar Regions	1980s–Present	https://nsidc.org/data/nsidc-0066	Several days
VIIRS (Suomi NPP/NOAA-20)	Snow Cover Binary EDR	375 m/Daily	Global (Land)	2012–Present	https://www.star.nesdis.noaa.gov/jpss/snow.php	3–6 h
VIIRS (Suomi NPP/NOAA-20)	Snow Cover Fraction EDR	750 m/Daily	Global (Land)	2012–Present	https://www.star.nesdis.noaa.gov/jpss/snow.php	3–6 h
H SAF H10	Snow Cover Extent from MSG-SEVIRI	5 km/Daily	Pan-European	Dec 2011–Present	https://hsaf.meteoam.it/Products/ProductsList?type=snow	3–6 h
H SAF H12	Snow Cover Fraction from AVHRR	1 km/Daily	Pan-European	Dec 2012–Present	https://hsaf.meteoam.it/Products/ProductsList?type=snow	3–6 h
H SAF H34	Snow Cover Extent from MSG-SEVIRI	5 km/Daily	Full MSG Disc	May 2019–Present	https://hsaf.meteoam.it/Products/ProductsList?type=snow	3–6 h
H SAF H35	Snow Cover Fraction from AVHRR	1 km/Daily	Northern Hemisphere	May 2019–Present	https://hsaf.meteoam.it/Products/ProductsList?type=snow	3–6 h
H SAF H43	Snow Cover Extent from MTG-FCI	2 km/Daily	Full MTG Disc	Dec 2024–Present	https://hsaf.meteoam.it/Products/ProductsList?type=snow	3–6 h

### EUMETSAT H SAF project and dedicated snow products

The European Organisation for the Exploitation of Meteorological Satellites (EUMETSAT, https://www.eumetsat.int/), established in 1986, operates Europe’s space-based system for weather, climate, and environmental monitoring in cooperation with international partners (Holmlund et al., 2025; Montagner, 2024). To support specialized applications, EUMETSAT coordinates eight Satellite Application Facilities (SAFs), each led by a Member State. These SAFs produce validated near real-time and offline data products, archived at both the EUMETSAT Data Centre and individual SAF portals (Forcada et al., 2006).

Among them, the H SAF (Support to Operational Hydrology and Water Management), active since 2005 and involving 12 European countries and ECMWF (https://hsaf.meteoam.it/), develops satellite-based products for precipitation, soil moisture, and snow monitoring. Its snow portfolio includes four product types: (i) snow extent (binary cover), (ii) effective snow cover (fSCA), (iii) snow status (dry/wet), and (iv) snow water equivalent (SWE).

#### H SAF snow cover extent products

**Figure 1:**
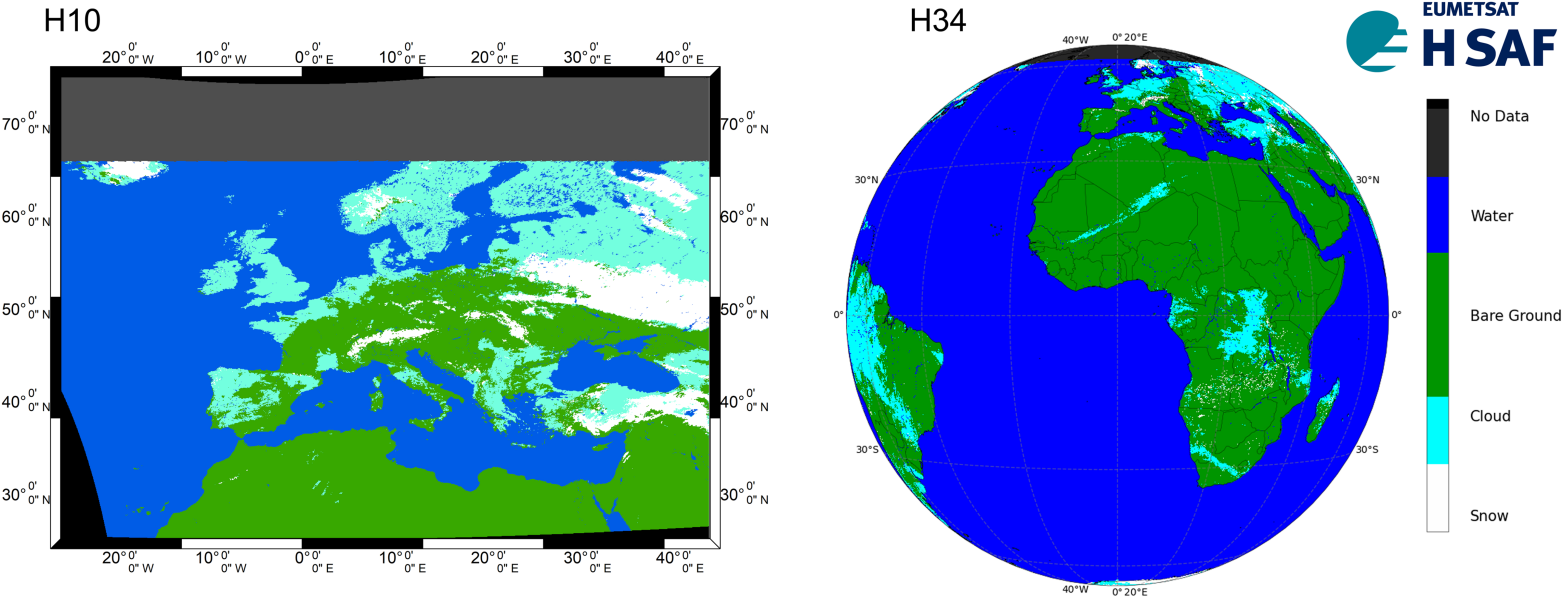
H10 (13 Feb 2022) and H34 (5 Feb 2025) SCE products.

The H10 product is a daily snow mask (*i.e*., SCE), created through snow detection using VIS/IR radiometry and multi-channel analysis with the Spinning Enhanced Visible and Infrared Imager (SEVIRI) instrument aboard Meteosat satellites (H-SAF_H10_PUM, 2018). It provides coverage across a pan-European region from 25°N to 75°N latitude (cf. Fig. 1). The SEVIRI instrument has an instantaneous field of view (IFOV) of 4.8 km at nadir, which increases to approximately 8 km in European coordinates, with a sampling rate of 3 km degrading to about 5 km. Each pixel is classified as either bare ground, cloud, snow, water, or no data. The H10 product consists of two main components: one for flat/forested regions, developed by the Finnish Meteorological Institute (FMI), and another for mountainous regions, developed by the Middle East Technical University (METU). Both parts were integrated into the operational environments of FMI and the Turkish State Meteorological Service (TSMS) by 2007. The data from both parts are merged at FMI using a mountain mask (H-SAF_H10_ATBD, 2011).

The H34 SCE product, which has been available since October 2017 as the successor to the H10, follows a similar generation chain. It utilizes the SEVIRI instrument on Meteosat satellites for daily snow detection *via* VIS/IR radiometry and multi-channel analysis. The product is produced using two independent algorithms—one for flat/forested areas (developed by FMI) and one for mountainous areas (developed by METU and executed at TSMS). The data from both algorithms are merged at FMI using a specific mask to combine information from both flat/forest and mountainous regions (H-SAF_H34_PUM, 2023). H34 offers an enhanced spatial domain, covering the full Meteosat Second Generation (MSG) disk with a horizontal resolution of ~5 km (cf. Fig. 1).

### The main motivation and the objectives of the study

The launch of the Meteosat Third Generation (MTG) satellite marks a major milestone in European Earth observation, significantly enhancing the capabilities previously offered by the MSG series (Antonio et al., 2009). Central to this advancement is the Flexible Combined Imager (FCI) sensor onboard MTG, which offers substantial improvements in spatial and temporal resolution—down to 0.5 km at nadir—and an expanded spectral range across 16 visible and infrared bands (Philippe et al., 2021). These features enable more frequent and accurate observations of dynamic surface processes, notably snow cover, which is particularly variable in mountainous regions.

Compared to MSG, which faced limitations in snow detection due to its coarser spatial detail, the MTG-FCI combination provides a promising platform for snow monitoring over complex terrains. Mountainous regions, in particular, pose unique challenges due to rapid elevation-induced variability, frequent cloud contamination, and mixed land cover. Accurate and timely snow-cover information in these regions is vital for hydrology, natural-hazard assessment, and climate monitoring.

Building upon this context, the present study is guided by a central research question:

How reliably can the newly developed MTG-based H43 product detect and map snow cover across complex mountainous environments when compared with previous SEVIRI-derived products and independent reference datasets?

The motivation for this study comes from the known spatial and spectral limitations of the earlier SEVIRI-based products (H10 and H34), which posed challenges in mapping snow accurately in rugged terrain. With its improved spatial resolution, stronger radiometric performance, and higher observation frequency, the new FCI sensor aims to overcome these limitations and enhance the operational use of EUMETSAT’s snow-monitoring system.

Within the framework of EUMETSAT’s H SAF programme, the new H43 operational SCE product has been developed using MTG-FCI data. H43 represents a continuation and enhancement of previous products such as H10 and H34, and aims to deliver improved snow detection over large and topographically diverse areas. This study provides the first evaluation of the H43 product, focusing on the period from 1 December 2024 to 28 February 2025, with an emphasis on performance in mountainous regions.

Validation is conducted through cross-comparison with the MODIS MOD10A1 Collection 6.1 NDSI snow-cover dataset, which offers high temporal and spatial availability, and with *in-situ* SD observations from WMO synoptic stations. Furthermore, terrain complexity is examined using elevation and slope aspect derived from MODIS DEM data, while land-cover effects are assessed using the MODIS MCD12Q1 product.

To address this research question, the study pursues three interrelated objectives:

- Product characterization and validation: Introduce the EUMETSAT H SAF H43 operational snow-mask product and evaluate its accuracy against MODIS-based NDSI snow maps and WMO *in-situ* SD measurements.
- Performance analysis across environmental gradients: Assess H43 performance across land-cover classes, elevation bands, and terrain aspects, with a specific focus on mountainous regions.
- Algorithm assessment and future improvement: Analyze the strengths and limitations of the H43 snow-detection algorithm and outline recommendations for refining operational snow-cover monitoring within H SAF.

Through these efforts, the study aims to provide an evidence-based assessment of the new H43 product’s capabilities, clarify its advancements over earlier SEVIRI-based products, and support the continued enhancement of continental- and global-scale snow-cover monitoring.

The remainder of this study is structured as follows: ‘Materials and Methods’ provides an overview of the MTG satellite system and the FCI sensor, including their technical specifications and advantages over previous MSG satellites. Additionally, this section introduces the datasets used in the validation process, including MODIS MOD10A1 NDSI snow cover C6.1 data and *in-situ* WMO SD measurements, as well as the validation methodology for the new H43 product. ‘Results’ presents the initial validation results for the H43 product, focusing on its performance across three geographic regions: the European Alps, Turkey, and Georgia (Caucasus). Finally, ‘Conclusions’ concludes the study, offering a discussion of the findings and potential future work related to the MTG-based snow cover products.

## Materials and Methods

### MTG satellite system and the FCI instrument

MTG satellite series represents a significant advancement in European geostationary meteorological observation. Developed by EUMETSAT in collaboration with ESA, MTG aims to ensure the continuity and enhancement of meteorological observations previously provided by the MSG series (Dieter et al., 2014). The MTG system comprises six satellites: four MTG-I (Imager) satellites and two MTG-S (Sounder) satellites (Holmlund et al., 2021). The MTG-I satellites carry the Flexible Combined Imager (FCI) and the Lightning Imager (LI), while the MTG-S satellites are equipped with the Infrared Sounder (IRS) and Copernicus Sentinel-4 payload for atmospheric composition monitoring (Donny et al., 2004). These satellites are designed to operate for at least 20 years, significantly improving weather forecasting, climate monitoring, and environmental applications.

The FCI onboard MTG-I satellites is a state-of-the-art instrument designed to provide high-resolution and high-frequency imaging of Earth’s atmosphere and surface. It is the successor to the SEVIRI onboard MSG satellites, offering significant enhancements in spatial, spectral, and temporal resolution. The key Improvements of FCI over SEVIRI can be listed as follows (Philippe et al., 2021; Yannig et al., 2015):

- Higher spatial resolution: Up to 0.5 km for VIS channels and 1.0 km for IR channels, compared to 1 km and 3 km in SEVIRI.
- Increased number of spectral channels: Expands from 12 bands (SEVIRI) to 16 bands (FCI), covering a broader range of the electromagnetic spectrum.
- Enhanced temporal resolution: Full-disk imagery every 10 min, with the capability to provide regional rapid scans every 2.5 min over specific areas.
- Improved radiometric performance: Higher radiometric sensitivity (*i.e*., 10-bit for SEVIRI, whereas 12-bit for FCI) and lower noise levels, enabling better atmospheric and surface feature detection.

FCI operates in two modes: (i) Full Disk Scan (FDS) Mode, capturing global Earth images every 10 min; and (ii) Rapid Scan (RS) Mode, providing regional images every 2.5 min over selected areas, improving the ability to monitor rapidly evolving weather phenomena such as thunderstorms and hurricanes. The FCI instrument covers a total of 16 spectral channels, categorized as follows: 4 VIS channels (0.4–0.8 µm); 2 NIR channels (0.8–1.6 µm); 8 IR channels (3.8–13.3 µm); and 2 high-resolution visible channels (HRV) (0.5 km resolution). The spectral channels of the Meteosat First Generation (MFG), MSG, and MTG satellites are compared in Table 2.

**Table 2:** The spectral channels used in MFG, MSG and MTG satellites (MVIRI: Meteosat Visible and InfraRed Imager, N/A: Not available) (Holmlund et al., 2021).

Spectral channel	MFG MVIRI		MSG SEVIRI		MTG FCI	
	Central wavelength (µm)	Spatial sampling (km)	Central wavelength (µm)	Spatial sampling (km)	Central wavelength (µm)	Spatial sampling (km)
VIS 0.4	N/A	N/A	N/A	N/A	0.444	1.0
VIS 0.5	N/A	N/A	N/A	N/A	0.510	1.0
VIS 0.6	0.7	2.5	0.635	3.0	0.640	0.5
VIS 0.8	N/A	N/A	0.81	3.0	0.865	1.0
VIS 0.9	N/A	N/A	N/A	N/A	0.914	1.0
NIR 1.3	N/A	N/A	N/A	N/A	1.380	1.0
NIR 1.6	N/A	N/A	1.64	3.0	1.610	1.0
NIR 2.2	N/A	N/A	N/A	N/A	2.250	0.5
IR 3.8	N/A	N/A	3.9	3.0	3.800	1.0
IR 6.3	6.1	5.0	6.2	3.0	6.300	2.0
IR 7.3	N/A	N/A	7.35	3.0	7.350	2.0
IR 8.7	N/A	N/A	8.7	3.0	8.700	2.0
IR 9.7	N/A	N/A	9.66	3.0	9.660	2.0
IR 10.5	11.5	5.0	10.8	3.8	10.500	1.0
IR 12.3	N/A	N/A	12.0	3.0	12.300	2.0
IR 13.3	N/A	N/A	13.4	3.0	13.300	2.0
Repeat cycle	30 min		15 min		10 min	

### H43 snow detection algorithm

**Figure 2:**
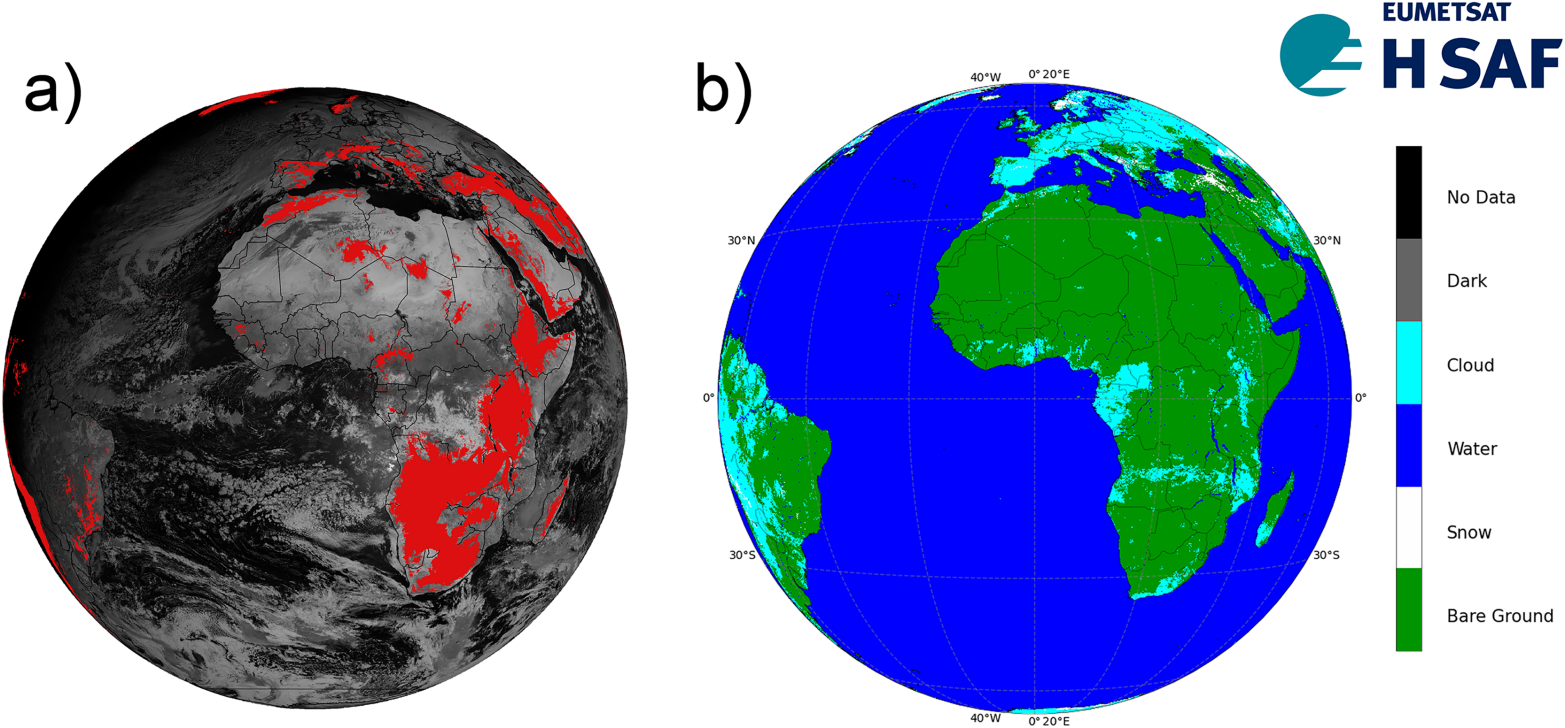
H43 mountain mask (A), and H43 SCE product on 13 March 2025 (B).

H43 Product (Snow detection (snow mask) by VIS/IR radiometry, cf. Fig. 2) is based on multi-channel analysis of the FCI instrument onboard Meteosat satellites. The FCI delivers data at a 1 km spatial sampling distance (resolution) at nadir (sub-satellite point the center of the disc). These figures degrade over Europe to 2–4 km. The observing cycle (10 min) enables continuous monitoring of the cloud situation, searching for time instants of cloud-free conditions in each time interval (*e.g*., 24 h).

Snow recognition in mountainous regions utilizes spectral thresholding methods applied to sub-pixel-scale MTG-FCI images. While the algorithm is developed to cover the full MTG disk extent, snow detection is specifically implemented in mountainous areas. A region is classified as mountainous if it meets any of the following conditions: the mean altitude exceeds 1,000 m; or the mean altitude exceeds 700 m and the standard deviation of the slope is greater than 2 degrees; or the mean altitude variation, defined as the difference between the maximum and minimum altitude within the mesh, exceeds 800 m and the mean altitude exceeds 500 m.

The snow mapping algorithm is a modification of the binary snow mapping method developed for MSG-SEVIRI by Sürer & Akyürek (2012), and Sürer, Parajka & Akyürek (2014). This algorithm utilizes top-of-atmosphere (TOA) values directly, without applying atmospheric correction to the images. The algorithm’s structure is based on the distinct spectral characteristics of clouds, snow, and land, which were determined through the subjective classification of known snow-cover features in MTG-FCI images. Threshold values for mountainous areas were derived from RGB composite images utilizing VIS 0.6 (centered at 0.64 µm), IR 1.6 (centered at 1.61 µm), IR 3.8 (centered at 3.8 µm), and IR 10.5 (centered at 10.5 µm). First, cloud discrimination was performed to ensure only cloud-free pixels were considered for snow and land classification. To remove cloud-covered pixels, the Cloud Mask (CMa) and Cloud Type (CT) products (Derrien, 2014) from the Nowcasting Satellite Application Facility (NWC SAF) were used. The cloud-filled class (pixel value 3) from CMa, indicating opaque clouds completely covering the IFOV, was applied. For cloud type discrimination, high opaque stratiform, very high opaque stratiform, and high semi-transparent thick clouds were excluded.

The NWC SAF Snow CMa and CT products were originally developed for SEVIRI, the instrument onboard MSG, and were only available for the period during which the H43 product was generated. Since SEVIRI’s native resolution at nadir is 3 km, its data was resampled to match FCI’s 2 km native resolution using nearest-neighbor interpolation. Additionally, SEVIRI’s 15-min temporal resolution required adaptation to FCI’s 10-min observation cycle. To achieve this, missing FCI time slots were mapped by repeating SEVIRI images, aligning 10- and 20-min SEVIRI images with 15-min timestamps and 40- and 50-min SEVIRI images with 45-min timestamps.

The algorithm begins by identifying snow pixels based on their high reflectance in the visible spectrum, selecting pixels with reflectance values greater than 0.3. Since snow exhibits low reflectance in the middle infrared and high reflectance in the visible range, a spectral index is used to enhance the distinction between snow-covered surfaces and other land types. A method similar to that of Dozier (1989) is applied, where the snow index (SI) is calculated as the ratio of IR1.6 to VIS0.6. Pixels with IR1.6/VIS0.6 values below a fixed threshold of 0.8 are classified as snow. To refine the selection, pixels with a low sun zenith angle (SZA) are filtered out, ensuring only those with an angle greater than 5° are retained. Additionally, a temperature threshold is applied to confirm snow-covered pixels. Since snow cannot exceed the freezing point, pixels with temperatures below 288 K in IR10.5 are accepted, following the approach of Romanov et al. (2003). This algorithm is applied to each 10-min MTG-FCI dataset between 06:00 and 16:00 UTC, generating 60 individual images per day for snow detection.

**Figure A1:**
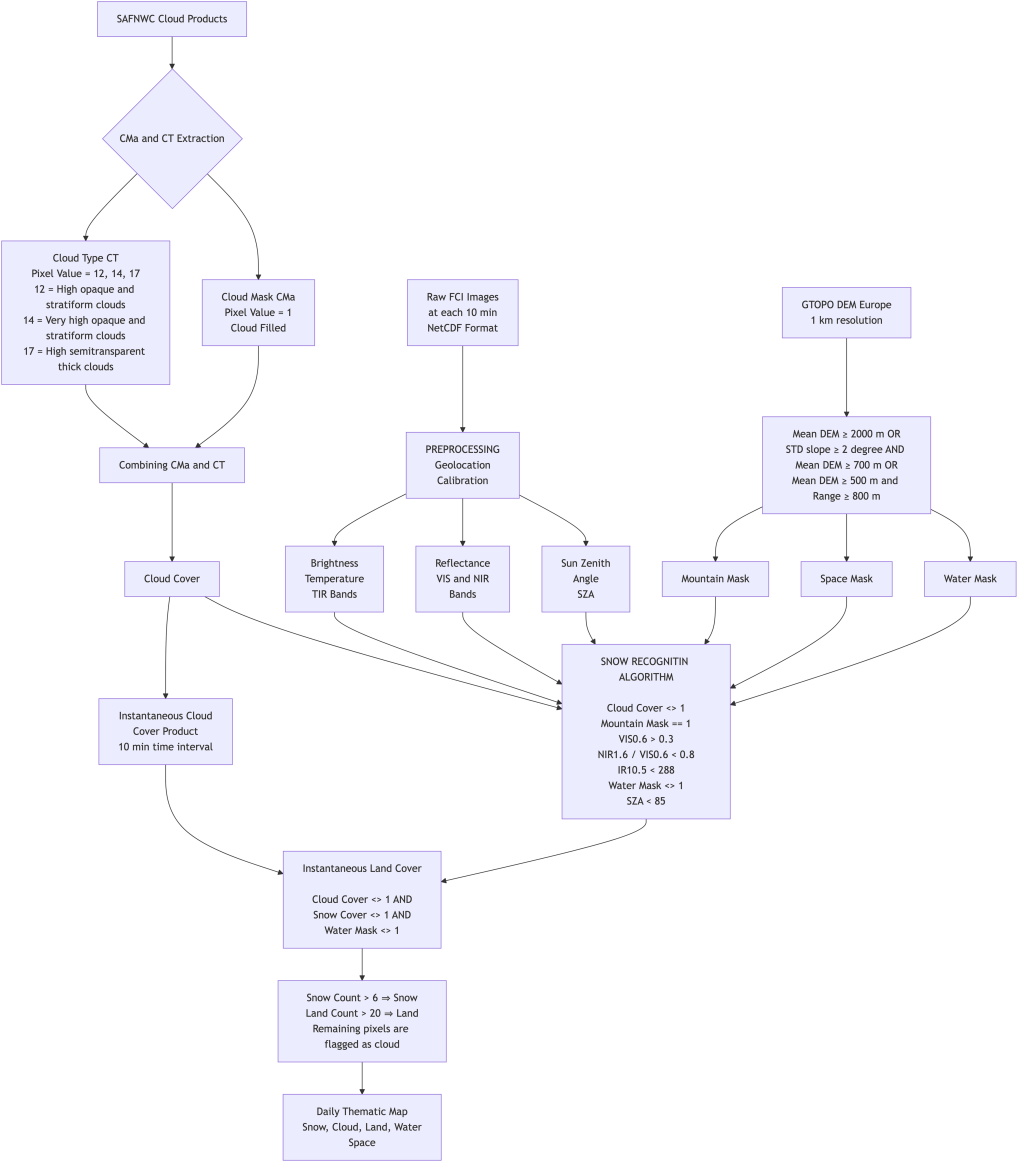
Flowchart illustrating the main processing steps of the H43 algorithm, including input data ingestion, cloud and snow discrimination, and generation of the final snow cover extend product.

After obtaining snow-cover maps for each individual 10-min image, a daily snow-cover map is generated by aggregating the data. For each classification category, the number of occurrences is counted across the 60 daily images. The snow pixels are classified by accepting pixels having at least six snow counts among the images. For cloud classification, the number of available measurements is determined for each pixel, accounting for SZA limitations. A threshold is set at 60% of the available zenith measurements, and pixels exceeding this threshold in cloud counts are labeled as cloud. Pixels with no available measurements due to sun zenith angle constraints are categorized as dark pixels. The final output is a daily thematic map with a 2-km spatial resolution, consisting of five distinct classes: snow, cloud, bare ground, water, and dark pixels. The algorithm flowchart of H43 is given in Figure A1 in the appendix.

### Spatial domain for the validation

The validation of the H43 SCE product is performed over three distinct geographic regions: the European Alps, Turkey, and Georgia-Caucasus. These areas have been selected to encompass a diverse range of topographic and climatic conditions, ensuring a comprehensive evaluation of the product’s performance. The classification of flat and mountainous terrain is based on the H43 mountain mask, while the percentage of water area is determined using the H43 water mask.

**Figure 3:**
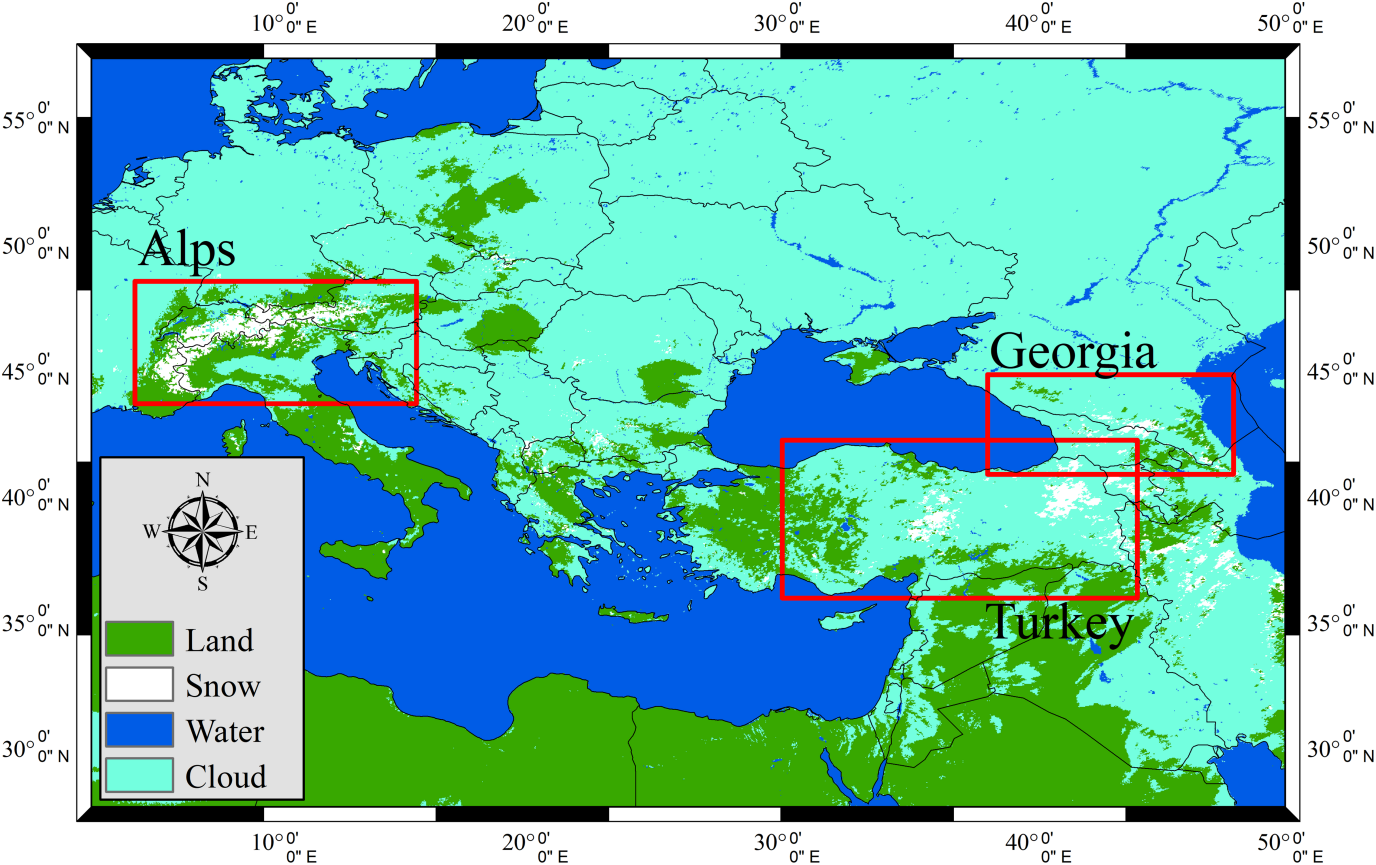
Validation regions over H43 SCE product on 9 January 2025.

The European Alps region extends between 48° 30′ 22.29″ N (top) and 43° 39′ 7.53″ N (bottom), and from 5° 6′ 30.21″ E (left) to 16° 17′ 30.69″ E (right), covering approximately 445,470 km^2^ (cf. Fig. 3). The region features a temperate alpine climate, where snowfall is frequent in winter, and seasonal snow cover significantly influences water resources (Gobiet et al., 2014). A total of 37% of the area is mountainous, 51% consists of flat terrain, and 12% is classified as water. The maximum elevation reaches 4,572 m, while the mean elevation is 1,607 m, indicating the presence of extensive high-altitude snow-covered regions. Snow cover in this region is subject to complex interactions with topography, making it an important test area for satellite-based retrievals.

The second validation region, Turkey, covers a vast area of 811,774 km^2^, ranging from 42° 12′ 2.24″ N (top) to 35° 55′ 7.84″ N (bottom), and from 30° 49′ 49.31″ E (left) to 44° 56′ 26.05″ E (right) (cf. Fig. 3). The region experiences a continental and mountainous climate, with harsh winter conditions in elevated areas and semi-arid characteristics in lower elevations (Turkes, 2020). The topographic distribution indicates that 61% of the region consists of mountainous terrain, 25% is flat land, and 14% is classified as water. The maximum and mean elevation values are 4,711 m and 1,553 m, respectively, indicating the dominance of mid- to high-altitude terrains. The presence of variable snowpack accumulation and frequent cloud cover makes it a critical region for validating H43’s detection capabilities.

The third region is mainly composed of the entire Georgia, south-west corner of Russia, and north-west portion of Azerbaijan, bounded by 44° 47′ 39.24″ N (top) and 40° 50′ 39.58″ N (bottom), and by 38° 59′ 30.90″ E (left) to 50° 0′ 31.76″ E (right), spanning an estimated 382,225 km^2^ (cf. Fig. 3). It is characterized by a humid continental to alpine climate, with cold, snowy winters and variable snow accumulation patterns influenced by the rugged Caucasus Mountains (Loveluck et al., 2024). The maximum elevation reaches 5,450 m, while the mean elevation is 1,637 m, making it one of the most rugged validation regions. Approximately 38% of the land area is mountainous, 37% is classified as flat terrain, and 25% is covered by water bodies. Snow retrieval in this region is particularly challenging due to steep terrain and mixed land cover types.

By including these three geographically distinct regions in the validation framework, this study ensures that the H43 product is evaluated under a wide range of environmental conditions. From rugged mountainous zones to moderately elevated terrains, this regional approach enables a comprehensive assessment of the product’s accuracy and operational value across varying landscapes and climate regimes.

### The reference snow datasets

Validating coarse-resolution snow cover products is challenging since there is no perfect “ground truth”. Both *in-situ* observations and higher-resolution satellite-derived snow maps (*i.e*., cross-sensor validation) provide useful reference data, but each has strengths and limitations (Kuter, Bolat & Akyurek, 2022). The accuracy of validation depends on how well the reference data matches the spatial and temporal scales of the satellite product being evaluated (Brown & Armstrong, 2008; Hall et al., 2019).

Ground-based measurements from meteorological stations and SD networks are often considered reliable because they provide direct observations of snow presence. These datasets cover long time periods, making them useful for evaluating seasonal and yearly snow trends (Avanzi et al., 2023; Orsolini et al., 2019). However, *in-situ* measurements only represent single points, while satellite observations cover large areas (Dong, 2018). This mismatch is a major limitation, especially in areas where snow cover changes over short distances, such as in mountains or forests (Gafurov et al., 2015; Hall et al., 2019). Another issue is that station networks are often sparse, especially in remote or high-altitude regions where snow is common. When station data is interpolated to estimate snow cover over a larger area, errors can occur, reducing reliability (Brubaker, Pinker & Deviatova, 2005; Hall et al., 2019). This means that unless there is a high density of stations, *in-situ* validation may not fully capture snow cover patterns observed by satellites.

A more spatially complete validation approach is cross-sensor validation, where snow maps from higher-resolution satellites are used as reference data. This method allows for a direct comparison of snow cover patterns over large areas, avoiding the point-based limitations of *in-situ* data. Satellite-derived reference snow maps can show small-scale snow distribution, making them valuable for evaluating coarse-resolution products (Hall, Riggs & Salomonson, 1995; Piazzi et al., 2019; Rittger, Painter & Dozier, 2013). However, cross-sensor validation also has challenges. High-resolution satellite data often has a shorter time record than *in-situ* observations, limiting long-term validation studies. In addition, both low- and high-resolution satellite snow products share some common errors, such as difficulty detecting snow in forests, cloud contamination, and differences in viewing angles (Dong, 2018; Gafurov et al., 2015). This means that even when comparing different satellite products, some biases may remain. Previous studies have shown that even within the same sensor family, changes in spatial resolution and processing methods can cause differences in results (Hall & Riggs, 2007; Hüsler et al., 2012).

Recognizing these challenges, an independent test dataset combining both *in-situ* measurements and higher-resolution reference snow maps has been designated for a comprehensive performance assessment of the H43 product, as well as H34. This combined approach ensures a more reliable and balanced evaluation, addressing the limitations of each method while improving the validation framework.

#### MODIS-derived reference snow maps

The validation of the H43 SCE product is conducted using reference snow cover maps derived from the MODIS MOD10A1 NDSI Collection 6.1 dataset (Riggs, Hall & Román, 2019). MOD10A1 is a daily global snow cover product generated from MODIS instrument onboard NASA’s Terra satellite. The MOD10A1 snow cover dataset has undergone extensive validation against both *in-situ* measurements and satellite-based reference datasets across a wide range of geographic settings, including complex mountainous terrains. Numerous studies have demonstrated its effectiveness, reporting accuracy levels between 77% and 100% (Gascoin et al., 2015; Klein, Barnett & Lee, 2003; Parajka & Blöschl, 2006; Tekeli et al., 2005). While some uncertainties arise due to cloud contamination, as well as minor accuracy reductions linked to snowpack properties and variations in illumination conditions (Brubaker, Pinker & Deviatova, 2005; Crawford, 2015), MOD10A1, with its 500 m spatial resolution, can be regarded as a well-established dataset for snow detection across diverse landscapes and climatic conditions. Given that this initial validation spans three winter months (December 2024–February 2025), MODIS provides the most suitable reference due to its daily temporal coverage and moderate 500 m spatial resolution, offering dense temporal sampling consistent with the period analyzed by the H SAF products.

In this study, reference snow cover maps are generated by applying a threshold of NDSI ≥ 0.4 to classify snow-covered pixels. This threshold has been widely adopted in the remote sensing community as a standard baseline for snow detection (Hall et al., 2002; Klein, Barnett & Lee, 2003). The threshold of 0.4 has demonstrated robust performance across diverse geographic and climatic settings and forms the foundation of the NASA MODIS snow algorithm. While several studies have explored the possibility of regionally optimized or dynamic NDSI thresholds to account for land cover heterogeneity, atmospheric conditions, or snow grain size (*e.g*., Gascoin et al. (2015), and Parajka & Blöschl (2006)), the universal threshold of 0.4 remains a practical and well-validated choice for large-scale, multi-region analyses. Given the wide spatial coverage of the current study and the need for consistency across three distinct mountainous regions, the use of the established 0.4 threshold ensures comparability and aligns with standard practices in operational and research-based snow mapping.

To generate the reference snow cover maps for validating H43, a systematic re-gridding and aggregation approach is employed to align the higher-resolution MODIS data with the coarser H43 (~2 km) spatial resolution. The methodology follows these steps: Each MODIS pixel with an NDSI ≥ 0.4 is classified as snow, while pixels with NDSI < 0.4 are labeled as non-snow. MODIS snow classifications are then aggregated over the exact footprint of each corresponding H43 (~2 km) pixel. The most frequently occurring class within the footprint (snow, non-snow, cloud, water, unclassified, no data) is assigned as the label for the corresponding H43 pixel. In cases where equal numbers of snow pixels and pixels with labels other than snow exist within the footprint, the pixel is always labeled as snow to ensure that snow presence is not underestimated.

**Figure 4:**
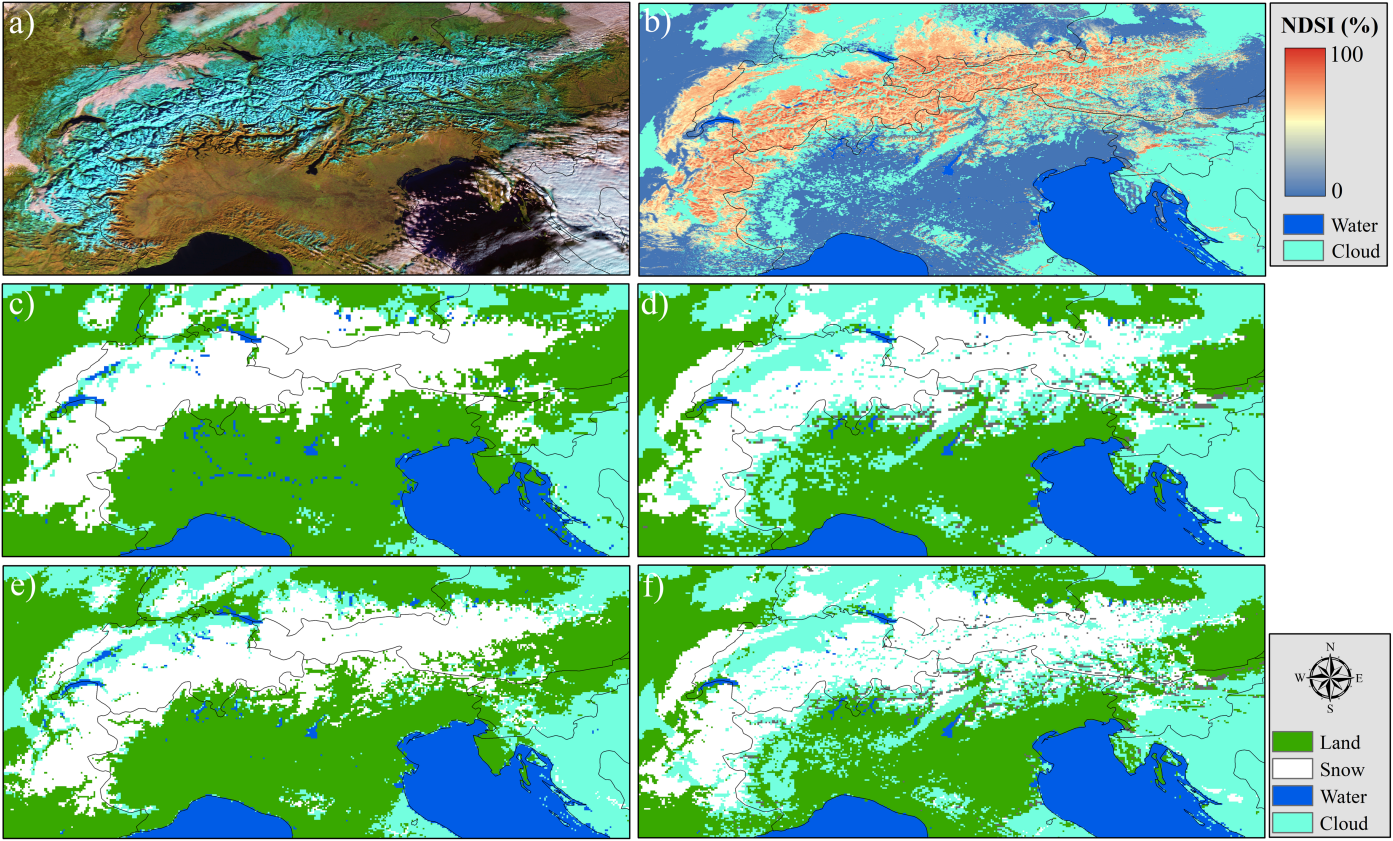
European Alps on 25 December 2024: (A) MODIS false color RGB, (B) MODIS NDSI snow cover, (C) H34 product, (D) MODIS NDSI-derived reference snow cover map for H34, (E) H43 product, (F) MODIS NDSI-derived reference snow cover map for H43.

This method enables a robust pixel-wise validation framework, ensuring that the reference dataset accurately represents the ground truth snow conditions for evaluating the performance of the H43 product. In addition to validating H43, reference snow cover maps are also produced for H34 to facilitate a direct intercomparison between these two products as applying the same approach in H43, yet taking the H34’s footprint into account (*i.e*., (~5 km). This intercomparison provides further insights into the improvements introduced in H43 and allows for a more comprehensive evaluation of the evolution of snow cover detection capabilities within the H SAF project. A set of SCE reference images for both H34 and H43 over the European Alps is illustrated in Fig. 4.

#### WMO *In-situ* snow depth dataset

**Figure 5:**
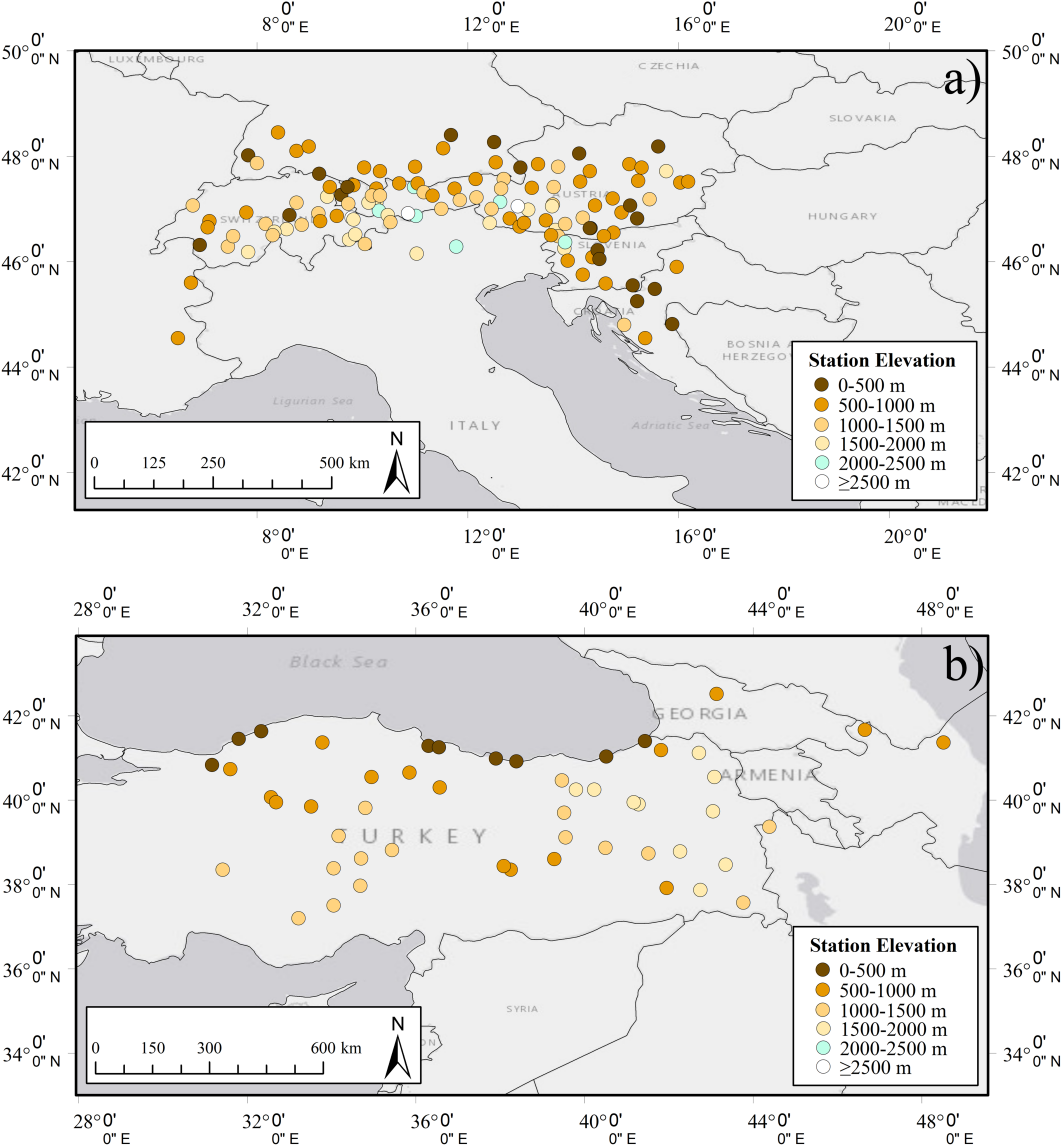
The locations of *in-situ* synoptic stations used in the study: (A) Alps, (B) Turkey and Georgia.

*In-situ* datasets consist of the observations from 176 synoptic stations (Alps: 125, Turkey: 48, Georgia: 3) from the Regional Basic Synoptic Networks (RBSNs) of WMO. The stations within the RBSNs are strategically distributed to ensure comprehensive coverage and timely data exchange on a global scale. SD observations from synoptic stations were recorded at standard times—00:00, 06:00, 12:00, and 18:00 UTC—covering the period from 2010 to 2025. The daily mean SD for each station was determined by averaging the recorded SD values. The spatial distribution of the stations with respect to 500 m elevation ranges is given in Fig. 5. For the *in-situ* validation, each station record was classified as snow-covered when the measured snow depth (SD) was equal to or greater than 5 cm, and snow-free when SD < 5 cm. This threshold provides a conservative and scale-consistent definition of snow presence, minimizing the influence of shallow or patchy snow that may not be reliably detected at the ~2 km spatial resolution of the H43 product. The selected 5 cm cut-off is consistent with the WMO Guide to Instruments and Methods of Observation, Volume II–Measurement of Cryospheric Variables (WMO-No._8, 2024) and with recent validation studies (Avanzi et al., 2023; Dong, 2018; Hall et al., 2019; Piazzi et al., 2019). The number of stations according to 500 m elevation zones are given in Table 3.

**Table 3:** Number of *in-situ* stations in each elevation zone over the validation regions.

Elevation range (m)	Alps	Georgia	Turkey
0–500	21	0	9
500–1,000	48	3	13
1,000–1,500	29	0	16
1500–2,000	18	0	10
2,000–2,500	6	0	0
≥2,500	3	0	0

### Land cover and terrain-related data

The MODIS MCD12Q1 Land Cover Type product provides global land cover classification at 500-m spatial resolution, offering five distinct classification schemes to support diverse modelling and environmental monitoring needs. Among these, the International Geosphere-Biosphere Programme (IGBP) classification is the most widely used, consisting of 17 land cover types, including various forest classes, croplands, grasslands, wetlands, and urban areas (Friedl et al., 2010; Sulla-Menashe & Friedl, 2022). The product is derived from time series of MODIS surface reflectance data, incorporating a supervised classification approach enhanced by ancillary data such as land surface temperature and elevation. Annual composites are generated for each year, enabling long-term monitoring of land cover change. The MCD12Q1 dataset is processed by NASA’s Land Processes Distributed Active Archive Center (LP DAAC) and made publicly available *via* the Earthdata portal (https://lpdaac.usgs.gov/products/mcd12q1v006/). In this study, the IGBP scheme was selected as the base reference; however, to facilitate interpretation and reduce class fragmentation, similar classes were merged, resulting in a generalized 10-class land cover map (cf. Table 4).

**Table 4:** MODIS MCD12Q1 original land cover classes and the classes after merging.

Original classes	Classes after merging
Evergreen needleleaf forests	Evergreen forests
Evergreen broadleaf forests	
Deciduous needleleaf forests	Deciduous forests
Deciduous broadleaf forests	
Mixed forests	Mixed forests
Closed shrublands	Grasslands/Shrublands
Open shrublands	
Grasslands	
Croplands	Mixed agriculture
Urban and built-up lands	
Cropland/Natural vegetation mosaics	
Woody savannas	Savannas
Savannas	
Barren	Barren
Water	Water
Permanent wetlands	Permanent wetlands
Permanent snow and ice	Permanent snow and ice

On the other hand, elevation and slope aspect values extracted from the MODIS 1-km Digital Elevation Model (MODDEM1KM) were used to evaluate the sensitivity of snow detection performance in relation to topographic features. MODDEM1KM product includes elevation, slope, and aspect layers, supporting topography-dependent analysis in environmental and climate studies. Generated by the NASA MODIS Land Science Team, this dataset is based on multiple global elevation sources—including the USGS GTOPO30 and SRTM datasets—resampled and harmonized to match the MODIS grid structure (Wolfe, 2013). Its standardized projection and consistent data format make MODDEM1KM well suited for regional-to-global scale applications involving land surface classification, snow mapping, and hydrological modelling.

### Statistical metrics used in the validation

To assess the accuracy and reliability of the H43 and H34 SCE products, a quantitative validation was performed using two independent reference datasets: MODIS-derived snow cover maps and *in-situ* snow depth (SD) observations from WMO station networks. The evaluation framework is based on a binary error matrix (cf. Table 5), which allows a direct comparison between satellite-derived and reference datasets under clear-sky conditions. From this matrix, the following statistical metrics were calculated (Gao et al., 2010):

- Probability of Detection (POD): the fraction of actual snow cases correctly identified by the satellite product, defined as POD = A/(A + C).
- False Alarm Ratio (FAR): the fraction of pixels incorrectly classified as snow relative to all snow detections, calculated as FAR = B/(A + B).
- Overall Accuracy (ACC): the proportion of correctly classified snow and non-snow cases, given by ACC = (A + D)/(A + B + C + D).

**Table 5:** The binary error matrix.

		Reference data	
		Snow	No snow
***Satellite*** ***product*** ***(H34/H43)***	**Snow**	HITS (A)	FALSE ALARMS (B)
	**No snow**	MISSES (C)	CORRECT NEGATIVES (D)
	**Cloud**	SNOW UNDER CLOUD (E)	CLOUD OVER BARE GROUND (F)

These metrics were derived only from clear-sky pixels (A–D), excluding cloud-affected cases (E, F). The cells E and F represent cloudy pixels where the satellite could not perform a snow/no-snow classification—specifically, E corresponds to cloud-snow misses (*i.e*., the station reports snow, but the satellite pixel is cloud) and F to cloud-no-snow (*i.e*., the station reports no snow, but the satellite pixel is cloud).

For the MODIS-based validation, only clear-sky matchups (A–D) were used. In addition, for the *in-situ* comparison, a separate ratio was calculated to quantify the percentage of snow observations obscured by clouds, expressed as E/(A + B + C + D + E + F) × 100, where E represents cases in which snow is observed at the ground station but the corresponding satellite pixel is classified as cloud. This analysis was performed for the Alps and Turkey regions but not for the Georgia–Caucasus region, due to the limited number of *in-situ* observations available. The resulting metric provides valuable insight into the impact of cloud contamination on snow detection availability across different sensors and regions.

To assess the statistical robustness of the accuracy metrics, 95% confidence intervals (CIs) were computed for ACC, POD, and FAR metrics derived from the binary error matrix. Each metric was treated as a binomial proportion, and the corresponding CI was estimated. Specifically, the denominators of the respective metrics (total observations for ACC, snow observations for POD, and detections for FAR) were used to define the number of trials in the binomial formulation (Brown, Cai & DasGupta, 2001). The resulting CIs quantify the uncertainty associated with each metric and allow for more robust inter-comparisons between regions and products. Due to the limited number of *in-situ* observations available over Georgia, confidence intervals were not reported for this region.

**Figure 6:**
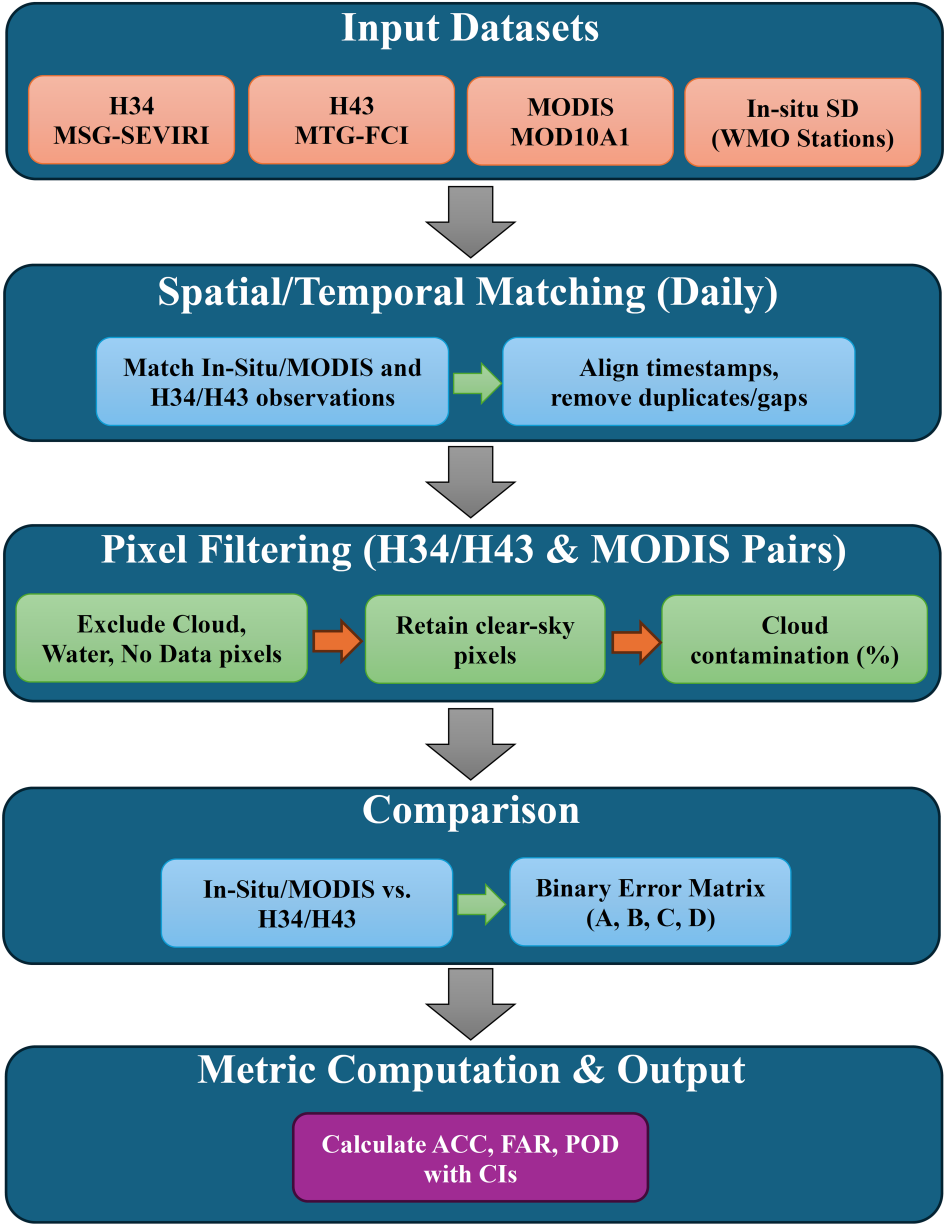
The flowchart of experimental design employed in the study.

The overall methodological workflow is illustrated in Fig. 6.

## Results

This section presents the validation results of the H43 SCE product, together with its predecessor (*i.e*., H34), across three geographic regions: the European Alps, Turkey, and the Georgia-Caucasus. The analysis is based on standard accuracy metrics—POD, FAR, and ACC—to evaluate the performance of both products under different environmental conditions. The validation is carried out at two levels: regional-scale analysis and land cover-based assessment. At the regional level, daily accuracy metrics are examined throughout the study period to understand how each product performs over time, including their sensitivity to seasonal changes and cloud contamination. In parallel, a land cover-based analysis is conducted using the land cover data obtained from the MODIS MCD12Q1 dataset, where similar land cover classes are grouped to improve interpretability. Additionally, accuracy metrics are also analyzed in relation to elevation and slope aspect, using data from the MODIS MODDEM1KM product, providing further insight into terrain-dependent performance.

### Regional-scale results

#### Results from the WMO snow depth dataset

This interpretation summarizes the snow detection performance of the H43 and H34 products based on *in-situ* observations from synoptic ground stations. The comparison includes the European Alps, Turkey, and the Georgia–Caucasus region. It should be noted that the number of *in-situ* observations is limited in each region and does not match the spatial and temporal coverage of MODIS-derived reference snow maps. Additionally, it should be noted that each *in-situ* snow depth observation was matched with the corresponding daily H43 and H34 pixel value at the same geographic location, ensuring a direct day-by-day comparison consistent with the temporal resolution of the satellite products. No temporal averaging or multi-day aggregation was applied, as the validation aimed to preserve the daily dynamics and reflect the true temporal behavior of the H43 and H34 products.

**Figure 7:**
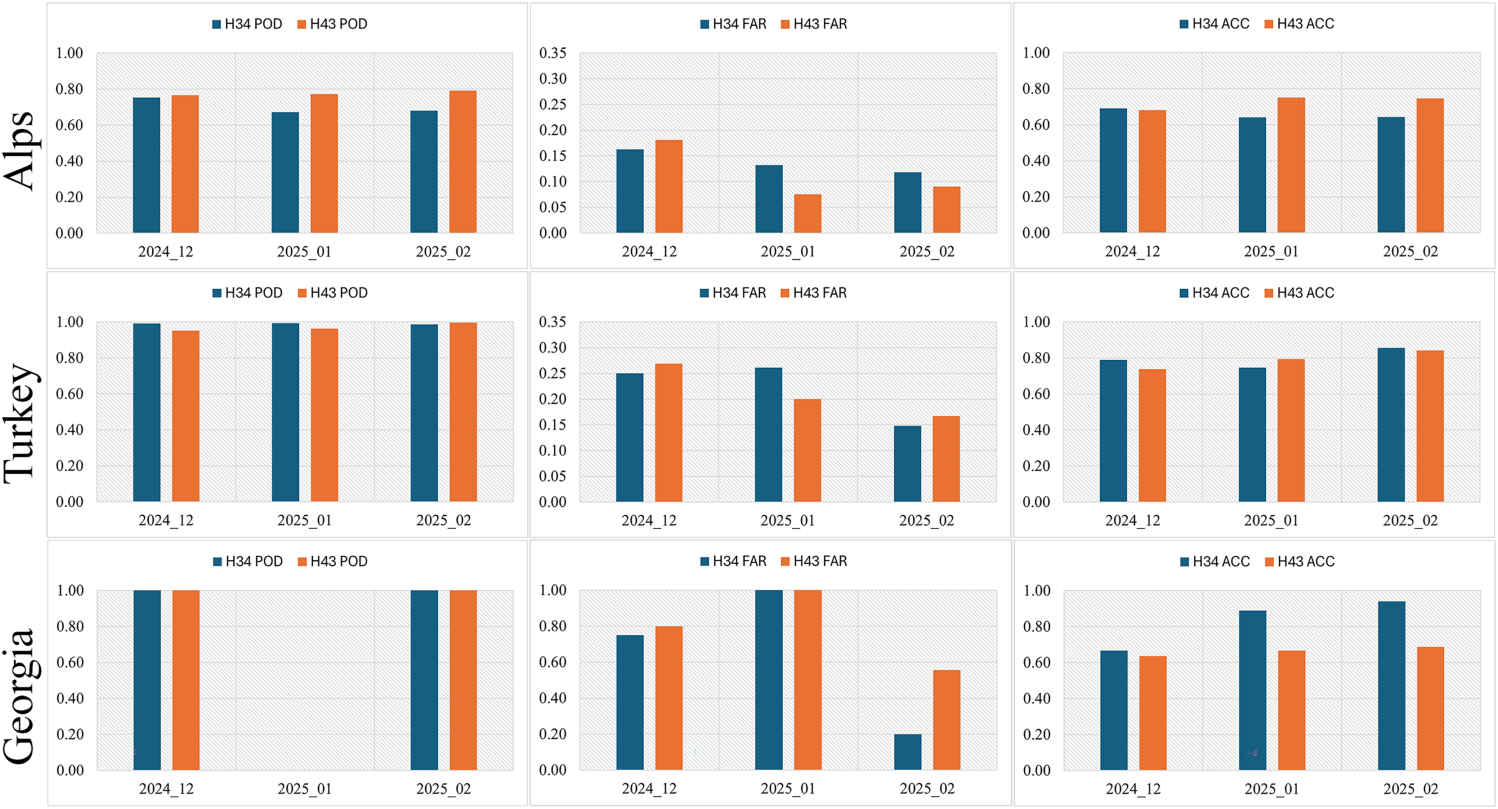
The statistical metrics from *in-situ* observations (Note: POD is undefined for January 2025 in Georgia region due to no true positives and false negatives in the observations).

Over the Alps region, the *in-situ* validation dataset consisted of approximately 2,500 snow depth (SD) observations gathered during the three winter months from up to 125 meteorological stations. These observations were used to generate pixel-level categorical comparisons between satellite-derived snow classifications and ground-based snow presence. While the detailed A, B, C, and D statistics are not tabulated, they underpinned the derivation of the performance metrics shown in Fig. 7. The number of satellite–*in-situ* comparisons (*i.e*., pixel–day pairs) per product and month varied between 550 and 1,056, providing a robust basis for evaluating detection performance.

The *in-situ* validation results over the Alps reveal that H43 delivers more reliable snow detection performance than H34 across all three winter months. The improvement is most evident in the POD, which was consistently higher for H43 (0.77–0.79) compared to H34 (0.67–0.75). In contrast, H34 exhibited higher miss rates (C) and FAR, particularly in December (H43 = 0.18, H34 = 0.16). Both products achieved similar overall accuracy (ACC) in December (H43 = 0.68, H34 = 0.69), but H43 demonstrated clearer improvements in the following months—by January, ACC increased to 0.75 for H43 *vs*. 0.64 for H34, supported by a reduced FAR (0.08 *vs*. 0.13). This trend continued into February, where H43 reached its highest performance (POD = 0.79, FAR = 0.09, ACC = 0.75), while H34 remained lower (POD = 0.68, FAR = 0.12, ACC = 0.64).

Overall, these results confirm that H43 provides more consistent and accurate snow detection over complex Alpine terrain, particularly during mid- and late-winter, when surface heterogeneity and persistent cloud cover pose challenges for satellite-based retrievals.

In Turkey, the *in-situ* SD observations contributed a total of ~650 satellite-to-ground match-ups spanning the three winter months. These were derived from measurements at up to 48 synoptic stations, with individual monthly station counts ranging from 14 to 47. Pixel-level classification comparisons were constructed based on the A, B, C, and D categories, reflecting agreements, false detections, omissions, and no-snow confirmations. While the full breakdown of these counts is not shown, they served as the basis for deriving the FAR, POD, and ACC metrics presented in Fig. 7.

The monthly validation metrics over Turkey indicate that both H34 and H43 demonstrate strong performance in detecting snow cover, but H43 shows overall improvements in key accuracy indicators, especially in the later months of the season. H34 already shows a very high POD across all 3 months (above 0.98), indicating a strong ability to correctly identify snow-covered areas. However, H43 exhibits even higher or comparable POD values in each month. Notably, in February 2025, H43 achieves a near-perfect POD of 0.9957, confirming its excellent snow detection capability. While H34 had a consistently high hit rate (A), H43 maintained similarly high hit rates and slightly improved detection performance, especially in months with increased snow cover extent.

H43 presents a modest reduction in false alarms compared to H34 in February, with a FAR of 0.1673 *vs*. H34’s 0.1480. However, in December and January, H43’s FAR is slightly higher, which may relate to localized cloud-snow confusion or terrain-induced variability in reflectance. These false alarms (B) were slightly more frequent in H43 early in the season but remained within acceptable limits and did not overshadow the gains in detection performance. In terms of overall classification accuracy, H43 demonstrates improved or comparable ACC values for each month. In January and February, H43 surpasses H34, achieving 0.7945 and 0.8423, respectively, compared to H34’s 0.7474 and 0.8571. The slight drop for H43 in February is negligible, particularly given its stronger POD and comparable FAR. The overall agreement with ground truth is clearly higher for H43 in January, which appears to be a transitional month in terms of snow conditions.

In the Georgia–Caucasus region, *in-situ* snow depth observations provided a limited but valuable dataset for validation, with nearly 65 match-ups collected from up to 3 stations. The resulting A, B, C, and D counts enabled basic agreement assessments despite the relatively small sample size. These values underpinned the monthly calculation of the FAR, POD, and ACC metrics (cf. Fig. 7). Due to data scarcity, the metrics derived for some months may reflect limited representativeness and should be interpreted with caution.

For H34, hit rates remained consistently high, with perfect POD values across all 3 months. However, the extremely small sample sizes magnified the impact of false alarms. In December 2024, H34 showed a high false alarm rate (FAR) of 0.75. January 2025 had zero hits and only one false alarm, resulting in undefined (NaN) POD. February showed the most reliable detection performance, with a lower FAR (0.20), supported by a more balanced distribution of hits and correct negatives.

For H43, POD remained 1.0 in all months, indicating successful identification of all snow cases in the limited sample. However, similar to H34, FAR values were elevated, particularly in December (0.80) and February (0.56), reflecting challenges in discriminating snow from snow-free conditions in such a small dataset. January again lacked any true positives, making it difficult to draw meaningful conclusions for that month.

The corresponding confidence intervals of ACC, FAR and POD metrics for H34 and H43 are given in Tables 6 and 7, respectively. For H34, high detection consistency was observed over Turkey, where POD values exceeded 0.98 with narrow CIs (±0.007–0.009), while lower and more variable POD values (0.68–0.75) were found over the Alps. FAR values of H34 decreased from December to February (0.25→0.15) in Turkey and remained low (0.12–0.16) in the Alps. ACC ranged between 0.74–0.86 in Turkey and 0.64–0.69 in the Alps, with CI widths typically below ±0.03.

**Table 6:** H34 confidence intervals (95%) for MODIS-based and *in-situ* validation metrics (LI: lower interval, UI: upper interval; boldface values indicate cases where the nominal range of 1.0 is exceeded).

Validation data source/Region/Time			POD			FAR			ACC		
			Mean	LI	UI	Mean	LI	UI	Mean	LI	UI
**MODIS**	**Alps**	**2024_12**	0.7899	0.7874	0.7924	0.0610	0.0594	0.0626	0.9148	0.9138	0.9470
		**2025_01**	0.7973	0.7945	0.8001	0.0530	0.0513	0.0547	0.9156	0.9144	0.9581
		**2025_02**	0.8226	0.8196	0.8256	0.0766	0.0744	0.0788	0.9315	0.9304	0.9578
	**Turkey**	**2024_12**	0.8769	0.8750	0.8787	0.0593	0.0579	0.0607	0.9463	0.9456	0.9224
		**2025_01**	0.8984	0.8970	0.8998	0.0624	0.0612	0.0635	0.9576	0.9571	0.9173
		**2025_02**	0.9168	0.9154	0.9181	0.0368	0.0358	0.0377	0.9572	0.9566	0.9043
	**Georgia**	**2024_12**	0.8983	0.8960	0.9006	0.0688	0.0669	0.0707	0.9210	0.9196	0.9158
		**2025_01**	0.8481	0.8454	0.8508	0.1144	0.1119	0.1168	0.9161	0.9149	0.9167
		**2025_02**	0.8564	0.8535	0.8593	0.0800	0.0777	0.0824	0.9026	0.9010	0.9325
***In-Situ***	**Alps**	**2024_12**	0.7540	0.7244	**1.0085**	0.1626	0.1358	0.1893	0.6921	0.6640	0.7202
		**2025_01**	0.6724	0.6413	**1.0071**	0.1322	0.1067	0.1576	0.6420	0.6131	0.6710
		**2025_02**	0.6798	0.6382	**1.0016**	0.1180	0.0852	0.1507	0.6440	0.6043	0.6837
	**Turkey**	**2024_12**	0.9911	0.9736	0.7836	0.2500	0.1802	0.3198	0.7889	0.7293	0.8485
		**2025_01**	0.9925	0.9780	0.7034	0.2611	0.1969	0.3253	0.7474	0.6856	0.8092
		**2025_02**	0.9874	0.9733	0.7213	0.1480	0.1062	0.1898	0.8571	0.8181	0.8962

**Table 7:** H43 confidence intervals (95%) for MODIS-based and *in-situ* validation metrics (LI: lower interval, UI: upper interval; boldface values indicate cases where the nominal range of 1.0 is exceeded).

Validation data source/Region/Time			POD			FAR			ACC		
			Mean	LI	UI	Mean	LI	UI	Mean	LI	UI
**MODIS**	**Alps**	**2024_12**	0.8138	0.8121	0.8155	0.0401	0.0391	0.0410	0.9296	0.9290	0.9303
		**2025_01**	0.8254	0.8234	0.8273	0.0437	0.0425	0.0448	0.9266	0.9258	0.9274
		**2025_02**	0.8798	0.8780	0.8815	0.0667	0.0654	0.0681	0.9486	0.9480	0.9492
	**Turkey**	**2024_12**	0.9199	0.9189	0.9209	0.0748	0.0738	0.0758	0.9496	0.9491	0.9501
		**2025_01**	0.9350	0.9342	0.9359	0.0628	0.0619	0.0636	0.9667	0.9663	0.9670
		**2025_02**	0.9654	0.9648	0.9660	0.0420	0.0414	0.0427	0.9704	0.9700	0.9707
	**Georgia**	**2024_12**	0.9248	0.9235	0.9262	0.0657	0.0644	0.0670	0.9322	0.9313	0.9331
		**2025_01**	0.8681	0.8662	0.8699	0.0830	0.0814	0.0845	0.9319	0.9311	0.9327
		**2025_02**	0.9238	0.9223	0.9252	0.0693	0.0679	0.0707	0.9321	0.9312	0.9331
***In-Situ***	**Alps**	**2024_12**	0.767	0.737	0.796	0.1813	0.1538	0.2089	0.6815	0.6528	0.7101
		**2025_01**	0.773	0.741	0.805	0.0752	0.0531	0.0974	0.7520	0.7213	0.7826
		**2025_02**	0.791	0.755	0.827	0.0905	0.0630	0.1179	0.7473	0.7110	0.7836
	**Turkey**	**2024_12**	0.951	0.913	0.989	0.2688	0.2001	0.3374	0.7394	0.6766	0.8021
		**2025_01**	0.963	0.927	0.999	0.2000	0.1312	0.2688	0.7945	0.7290	0.8601
		**2025_02**	0.996	0.987	**1.004**	0.1673	0.1232	0.2114	0.8423	0.8009	0.8837

The H43 product demonstrated slightly improved performance, with POD values reaching 0.95–0.99 over Turkey and 0.77–0.79 over the Alps, accompanied by narrower CIs (±0.015–0.018). FAR values were lower than those of H34, particularly over the Alps (0.09–0.18), while ACC values increased modestly to 0.74–0.84 in Turkey and 0.68–0.75 in the Alps.

Overall, both products exhibited high sensitivity (POD), but H43 showed slightly higher false alarm rates than H34, especially in February. The scarcity of *in-situ* observations in the region and the disproportionate effect of individual misclassifications contribute to erratic month-to-month variations in accuracy metrics. Despite these limitations, H43 consistently maintained 100% detection capability when snow was present, suggesting robustness in hit rate performance under data-constrained conditions. Future assessments in this region would benefit greatly from denser and more consistent ground station coverage to improve the statistical reliability of validation results.

Across Alps and Turkey regions, the analysis quantifying the percentage of snow conditions that could not be detected due to cloud cover under all-sky conditions (cf. Statistical Metrics used in the Validation) demonstrate the clear benefit of the higher temporal resolution provided by the Meteosat missions. Over the Alps, the percentage of snow observations missed due to cloud contamination was 28.4% for H34 and 30.4% for H43, compared with 47.1% for MODIS. In Turkey, similar trends were observed, with missed snow rates of 25.2% for H34, 25.7% for H43, and 48.9% for MODIS. These findings confirm that the frequent observations from MSG (every 15 min) and MTG (every 10 min) substantially reduce the snow data gaps due to cloud contamination, improving the temporal continuity of snow cover monitoring. The results are consistent with those reported by Sürer & Akyürek (2012) and Sürer, Parajka & Akyürek (2014), who demonstrated that merging multiple SEVIRI acquisitions per day significantly improves snow detection in mountainous areas by mitigating the effects of cloud and illumination variability. Overall, the present analysis shows that H43, derived from the MTG-FCI sensor, preserves the operational strengths of its predecessor while offering enhanced capability for near-real-time snow detection under variable atmospheric and illumination conditions.

In summary, the *in-situ* validation reinforced the improved capabilities of H43 over H34 across a range of geographic and climatic conditions, particularly in complex mountainous terrains and during periods of peak snow accumulation. While the results from WMO synoptic stations confirmed H43’s enhanced accuracy and detection reliability, the limited spatial and temporal density of *in-situ* observations—especially across under-monitored regions such as the Georgia–Caucasus—posed constraints on comprehensive performance assessment. To overcome these limitations, this study employed a cross-sensor validation framework by leveraging MODIS MOD10A1 NDSI-based reference snow cover maps, which offer superior temporal continuity, higher spatial coverage, and consistent data availability. Consequently, the combined use of satellite-based reference datasets and ground observations ensures a more complete and statistically meaningful assessment of snow detection performance.

#### Results from the MOD10A1 NDSI-derived reference dataset

The cross-sensor validation using MODIS-derived reference datasets provides a more robust and spatially comprehensive evaluation framework, as it incorporates a significantly larger number of daily observations in the error matrix. Both H43 and H34 were validated against daily MODIS MOD10A1 snow-cover maps using corresponding daily composite images, ensuring full temporal consistency and comparability between datasets. The high spatial resolution and daily temporal coverage of MODIS ensure broader geographic representation and improved sampling across diverse snow and land surface conditions. This comprehensive coverage enhances the statistical reliability of accuracy metrics, supporting a more meaningful comparison of snow detection performance between H34 and H43 products.

**Figure 8:**
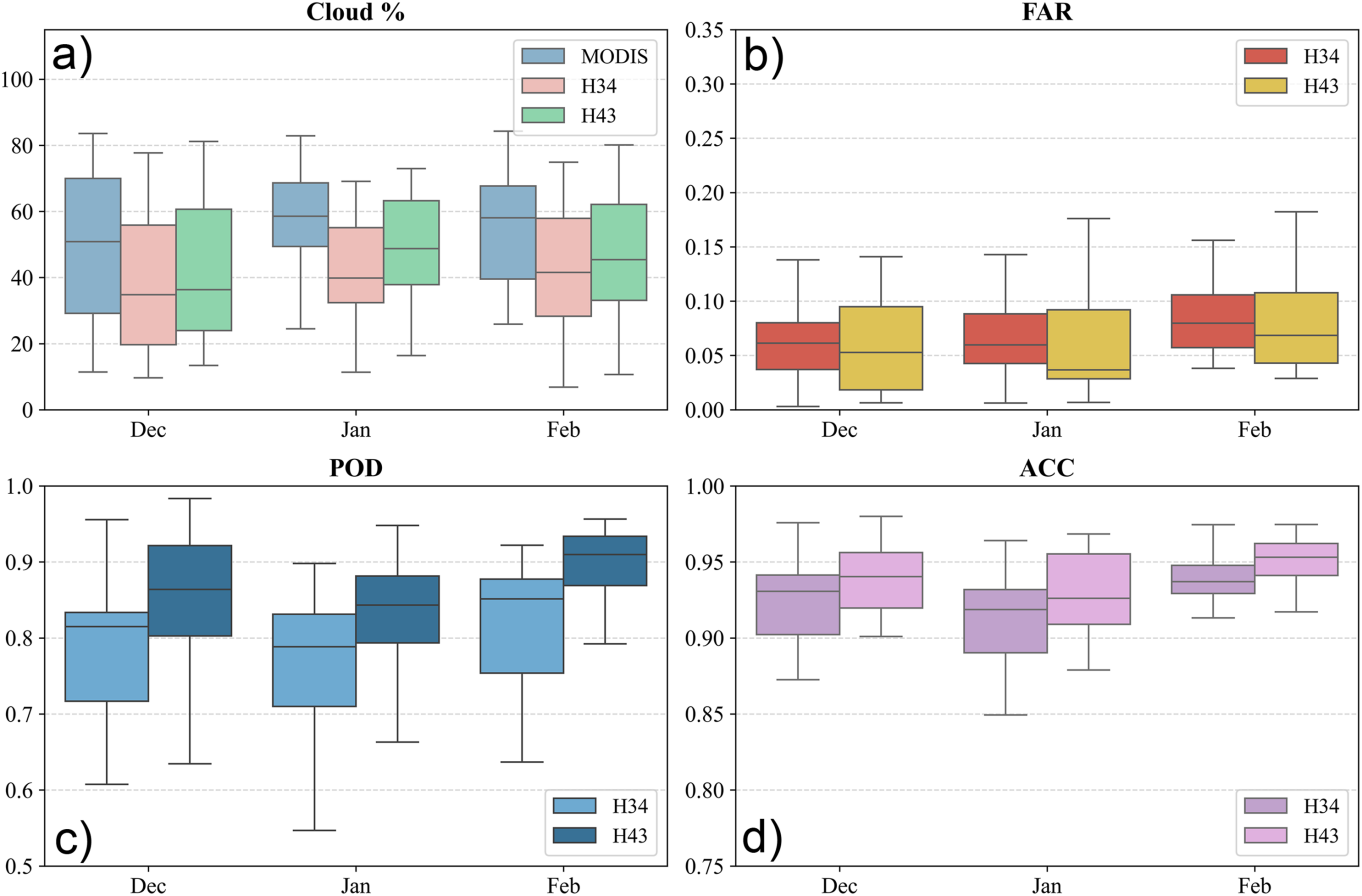
(A) Cloud cover percentage, (B) FAR, (C) POD, and (D) ACC metrics for the European Alps between 1 December 2024 and 28 February 2025.

The minimum, maximum and mean values of the statistical metrics over the European Alps is provided in Fig. 8, together with the cloud cover percentage. The results show that H43 consistently performs better than H34, with higher POD and ACC values, and a slightly lower FAR on most days. The lowest PODs were recorded in early December, likely due to increased cloud cover. Both products maintained high ACC overall, but H43 showed stronger alignment with the MOD10A1 NDSI reference dataset. Monthly analysis indicates that in December, both products had similar POD values near 0.77. In January, H43 improved significantly to 0.92, while H34 remained steady. By February, H43 reached its peak POD at 0.95, reflecting enhanced snow detection. FAR values were lower in December, suggesting fewer false positives early in the season. However, they rose slightly in January and February, likely due to changing snow conditions and melting events. Accuracy stayed consistently high, with H43 achieving the highest ACC of 0.97 in February. Cloud cover strongly influenced both products, lowering POD and ACC on heavily clouded days, though H43 proved more robust. The most cloud-affected periods were early December and mid-February, during which accuracy occasionally declined.

Overall, H43 showed better snow detection performance than H34, especially in terms of POD and ACC. H34 had more variability and a slightly higher FAR. Seasonal trends revealed that POD improved over time, with a clearer increase in H43. Although cloud cover remained a limiting factor, its impact was less noticeable in H43, suggesting that the updated algorithm handles cloud interference more effectively. These results underline the improved ability of H43 to detect snow cover in complex terrain such as the European Alps.

**Figure 9:**
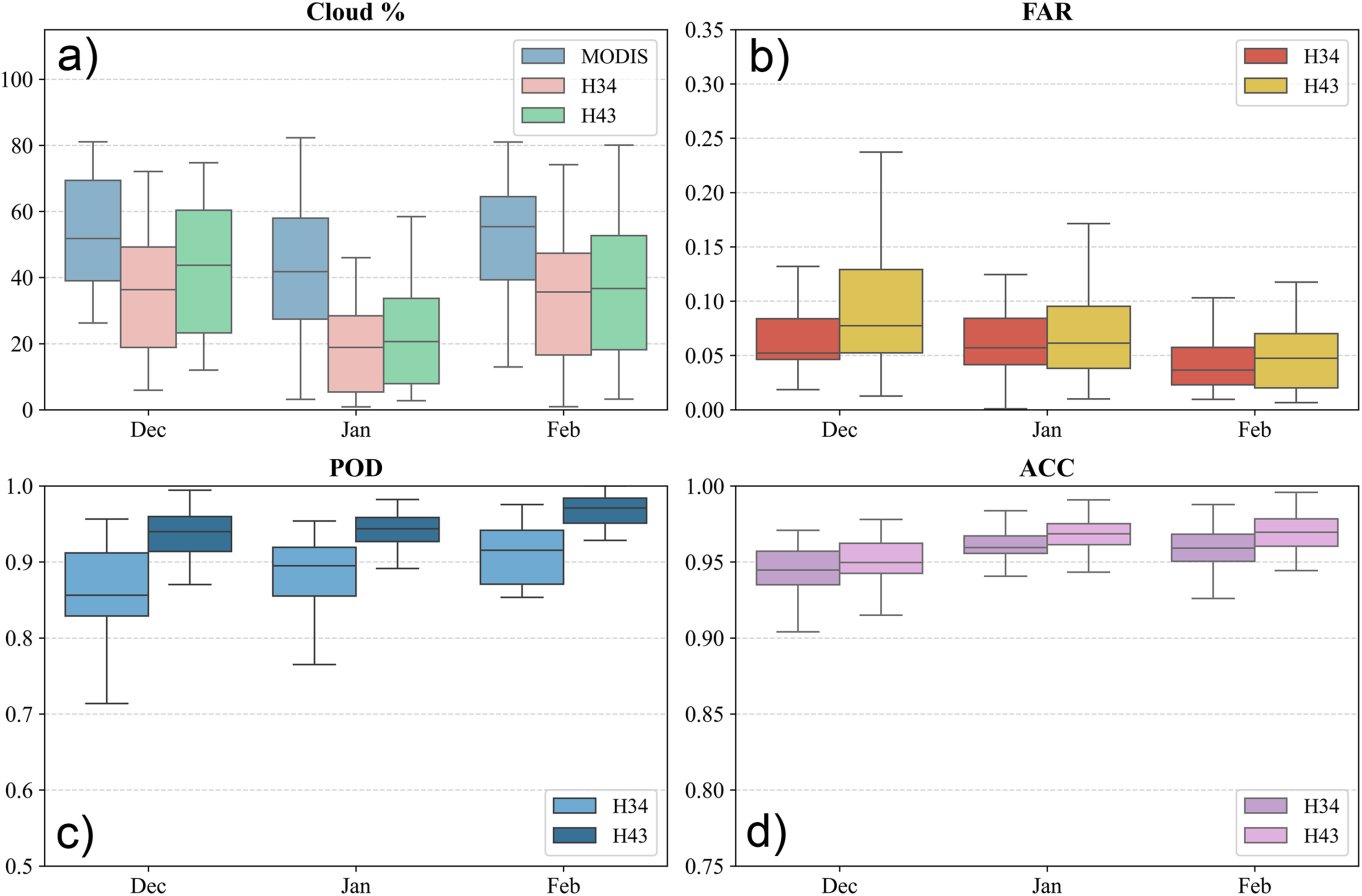
(A) Cloud cover percentage, (B) FAR, (C) POD, and (D) ACC metrics for Turkey between 1 December 2024 and 28 February 2025.

Over Turkey, H43 consistently outperformed H34 in detecting snow cover. The POD for H34 ranged from 0.615 to 0.976, with an average of 0.88. In comparison, H43 showed a higher and more stable range between 0.80 and 1.00, averaging 0.94 (cf. Fig. 9). The FAR for H34 varied between 0.0009 and 0.1701, while H43 ranged from 0.007 to 0.237. Despite the broader range, H43’s mean FAR was 0.070, only slightly higher than H34’s 0.060, indicating that the improved detection did not come at the cost of significantly more false alarms. In terms of ACC, H43 maintained stronger consistency, ranging from 0.857 to 0.996, compared to 0.892 to 0.987 for H34. The mean ACC was 0.96 for H43 and 0.95 for H34, highlighting H43’s improved performance in capturing snow distribution across Turkey.

Monthly analysis shows that snow detection improved from December to February. In December, the average POD was 0.858 for H34 and 0.931 for H43. By February, detection increased to 0.896 for H34 and 0.959 for H43. False alarm ratios stayed relatively stable, with minor changes in January likely due to changing snow conditions. Accuracy was highest in February when snow classification became more consistent. Cloud cover had a strong impact, especially in early December and mid-February, lowering accuracy for both products. However, H43 handled cloud interference better than H34, maintaining steadier performance. Overall, the results over Turkey confirm that H43 performs better than H34, offering higher detection rates and better accuracy, with only a small increase in false alarms. Seasonal changes affected both products, but H43 proved more effective, especially in cloud-affected conditions.

**Figure 10:**
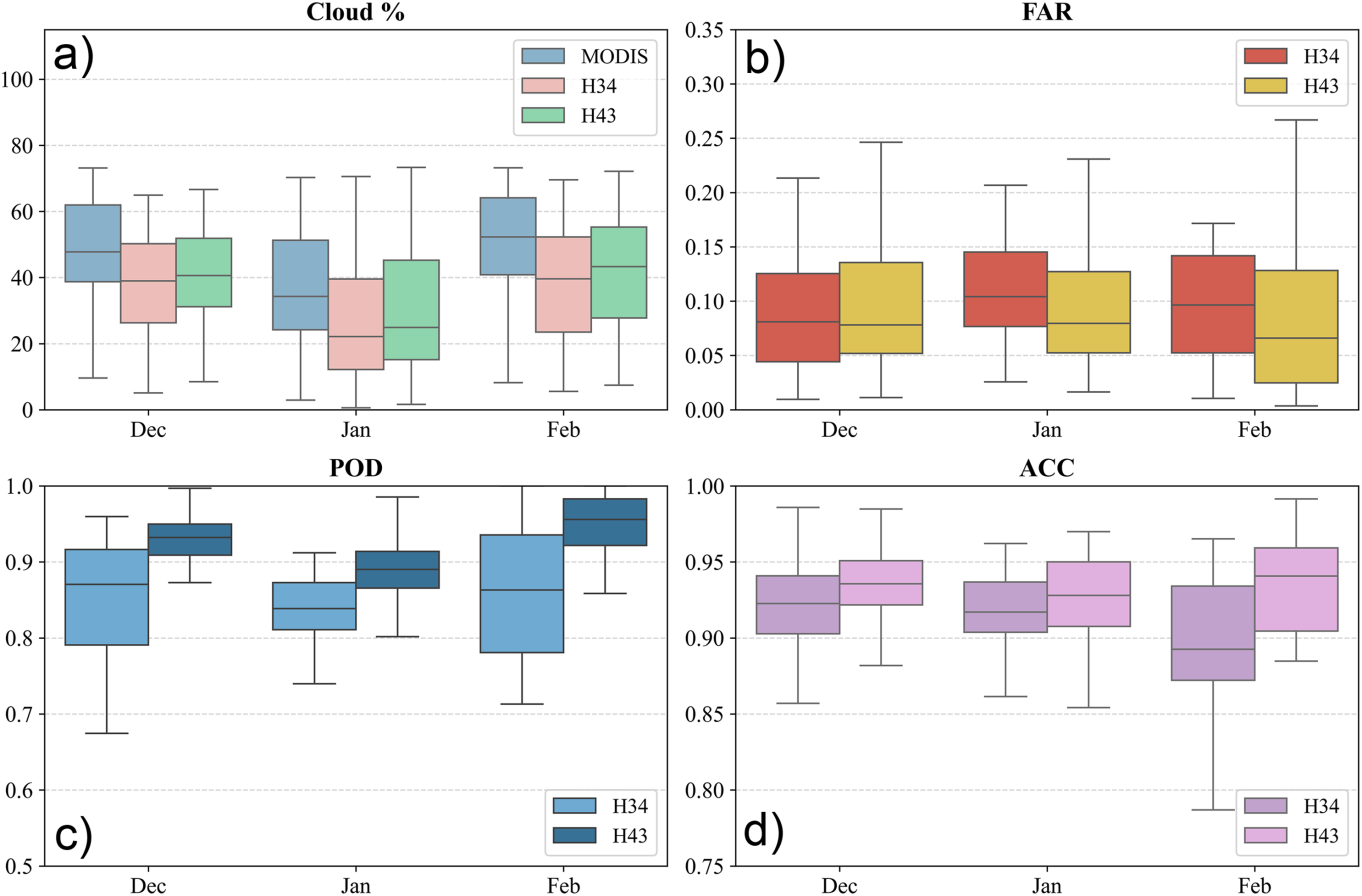
(A) Cloud cover percentage, (B) FAR, (C) POD, and (D) ACC metrics for Georgia-Caucasus between 1 December 2024 and 28 February 2025.

H43 generally showed better snow detection than H34 over the Georgia-Caucasus region, with higher POD and ACC values and only a slightly higher FAR. Both products performed better in the later months, with seasonal effects influencing detection. While cloud cover continued to impact accuracy, H43 proved more effective in handling it. Over the full period, H34 had a POD between 0.674 and 0.939, averaging 0.854, while H43 ranged from 0.712 to 0.955, with a slightly higher average of 0.858 (cf. Fig. 10). FAR values were similar overall: H34 ranged from 0.028 to 1.000, and H43 from 0.011 to 0.372, with mean values of 0.102 and 0.128, respectively, indicating a small increase in false positives for H43 under certain conditions.

H43 consistently achieved higher overall accuracy than H34, with ACC values ranging from 0.817 to 0.991, compared to 0.902 to 0.985 for H34. The mean ACC was 0.932 for H43 and 0.91 for H34, confirming H43’s stronger agreement with the reference data. Monthly trends show increasing POD for both products from December to February, with H43 improving more significantly. In December, average POD values were 0.854 for H34 and 0.923 for H43, rising in February to 0.858 and 0.937, respectively. FAR remained generally stable, though slightly higher in January—possibly due to transitional snow conditions. ACC followed a similar pattern, with the highest values in February when snow cover detection was more stable. Cloud contamination, especially in early December and late February, caused noticeable drops in POD and ACC. Still, H43 showed better resistance to cloud-related impacts.

The H43 product is based on blending 60 consecutive images per day, which is foreseen as an alternative to different filtering methods used for cloud reduction in optical remote sensing products. The results indicate that the blending of multiple observations during the day allows a significant cloud reduction over the three study regions.

The confidence interval (CI) analysis derived from MODIS-based validation provided a detailed assessment of the reliability and temporal stability of the H34 and H43 snow products (cf. Tables 6 and 7, respectively). For H34, POD values were consistently high across all regions, ranging from 0.79 to 0.92 with narrow confidence intervals (±0.001–0.002). The highest detection performance was observed over Turkey and Georgia, while slightly lower but stable POD values were recorded over the Alps. FAR remained low throughout the winter period, decreasing from approximately 0.06 to 0.04 in Turkey and maintaining comparable levels in Georgia and the Alps. ACC values were uniformly high for H34, typically exceeding 0.90 across all regions with very narrow confidence intervals (±0.0003–0.0008), confirming the statistical robustness of the validation results.

The H43 product demonstrated slightly improved detection and overall performance compared to H34. POD values increased to 0.92–0.97 in Turkey and Georgia, and to 0.81–0.88 over the Alps, accompanied by similarly narrow CIs (±0.001). FAR values for H43 remained low, generally between 0.04 and 0.07, indicating consistent discrimination of snow-free areas. Accuracy values exceeded 0.93 for all regions, with minimal seasonal variation and CI widths below ±0.0005. These results highlight the strong statistical stability of both products, with H43 exhibiting marginally higher accuracy and lower uncertainty than H34.

The comparison between MODIS-based and *in-situ* validations clearly demonstrates the effect of sample size on the statistical confidence of accuracy metrics. Owing to the substantially larger number of match-up observations in the MODIS-based validation, the CIs of all performance metrics (ACC, POD, and FAR) became significantly narrower compared to those derived from the limited *in-situ* dataset. This reflects the expected statistical behavior of binomial estimators, where larger sample sizes reduce the uncertainty of proportion-based metrics. Consequently, the narrower CIs obtained from the MODIS-based analysis indicate a higher level of statistical precision and reliability in the estimated accuracy values, whereas the wider intervals observed in the *in-situ* validation mainly arise from the smaller number of collocated snow observations.

#### Performance with respect to elevation and aspect

To complement the overall performance assessment, the spatial distribution of classification errors—specifically false alarms (*i.e*., B) and missed detections (*i.e*., C)—was further analyzed with respect to elevation zones and terrain aspect across the three validation regions. This terrain-based evaluation provides insight into the topographic dependencies of the H34 and H43 products, helping to identify systematic retrieval challenges associated with snow detection in varying altitudinal belts and slope orientations. Such analysis is crucial for improving algorithm robustness in complex mountainous environments.

All evaluations were performed using percentage values rather than raw pixel counts. This approach was necessary because the H34 and H43 products differ in spatial resolution. Direct comparison of raw pixel counts could lead to biased interpretations, as the total number of classified pixels per unit area is not the same between the two datasets. It was preferred to focus on false alarms (B) and misses (C) since they highlight two key sources of uncertainty: overestimation (false detection of snow) and underestimation (failure to detect existing snow). These two errors directly affect the usability of satellite snow products in hydrology, modelling, and snowpack monitoring. Additionally, the analysis focuses on elevation zones starting from 1,000–1,500 m, as snow cover below this threshold is typically rare and short-lived. Including lower zones could distort results due to limited observations and higher variability. By focusing on mid- to high-elevation ranges, the assessment better reflects product behavior under consistent snow conditions relevant for cryosphere monitoring.

**Figure 11:**
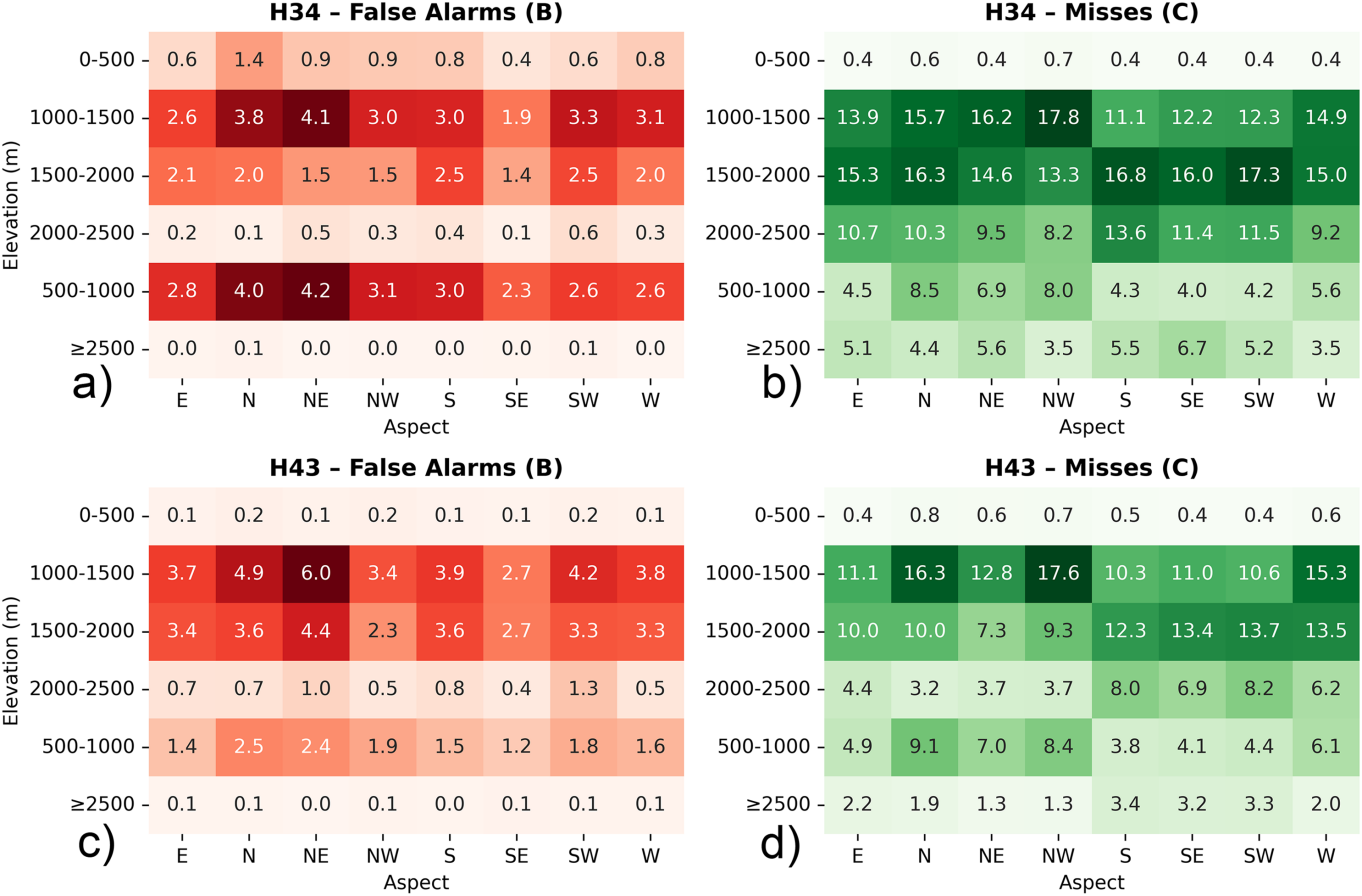
Heatmap diagrams of: (A) H34 false alarms, (B) H34 misses, (C) H43 false alarms, and (D) H43 misses, by elevation and aspect for Alps. Metrics are expressed as percentages to ensure consistency between H34 and H43 products with differing spatial resolutions.

For the European Alps, within the 1,000–1,500 m elevation band—where snow cover typically becomes more intermittent and sensitive to short-term meteorological variability—H34 and H43 snow products exhibit comparable behavior in terms of classification uncertainty, though with nuanced differences (cf. Fig. 11). Misses remain slightly higher in H34 (~1.58%) relative to H43 (~1.46%), indicating that H34 may occasionally overlook snow patches in areas with transitional snow cover or complex surface conditions. On the other hand, false alarms are marginally more frequent in H43 (~0.45%) than in H34 (~0.34%), suggesting a greater likelihood of overestimating snow presence under fluctuating radiometric or terrain-driven reflectance patterns. This elevation range, often characterized by fragmented snow fields and active melt cycles, appears to challenge both products, though H43 adopts a slightly more inclusive snow-mapping approach. Looking across directional slopes, north- and east-facing aspects tend to enhance these differences. H34 shows a moderate level of misses over shaded and potentially snow-retentive orientations, while H43 records somewhat increased false alarm levels in the same sectors. These distinctions highlight the varying sensitivities of the two products to topographic modulation of snow visibility—whether through early morning illumination, slope shading, or terrain-forced heterogeneity.

Both H34 and H43 snow cover products exhibit moderate levels of classification uncertainty in the 1,500–2,000 m elevation range, yet they differ in how that uncertainty manifests. H34 shows a higher rate of misses (~1.71%) compared to H43 (~1.24%), suggesting that it may apply more selective snow labeling in areas where snow conditions are variable or less clearly distinguishable. This could be influenced by partial snow coverage, shadow effects, or mixed-pixel complexities in mid-elevation terrain, where snowpack tends to be less stable than at higher altitudes. Conversely, false alarm rates are higher in H43 (~0.37%) than in H34 (~0.21%), indicating that H43 is more likely to misclassify snow-free surfaces as snow-covered in this elevation band. This behavior may reflect increased surface brightness, terrain reflectance anisotropy, or temporary snow events that lead to misclassification under certain illumination conditions. Aspect-based analysis reveals that north- and east-facing slopes show relatively higher rates of both misses and false alarms in H43, suggesting sensitivity to terrain-induced illumination effects or early morning solar exposure. H34, while showing lower false alarm percentages overall, tends to miss more snow presence across these aspects.

In the 2,000–2,500 m elevation range, H34 exhibits a higher average rate of misses (~1.16%) compared to H43 (~0.60%), indicating a more selective detection of snow-covered areas within this elevation band. This behavior may reflect the influence of residual cloud contamination, terrain-induced shading, or spectral confusion between snow and other high-reflectance surfaces such as rocks and glacier ice. In contrast, H43 demonstrates a more complete snow retrieval pattern, likely supported by its conservative thresholding strategy and possibly more consistent classification over mountainous terrain under clear-sky conditions. At the same time, H34 shows a higher false alarm rate (~0.74%) relative to H43 (~0.12%), suggesting that H34 is more prone to assigning snow labels to snow-free pixels, especially in radiometrically complex or geometrically challenging areas. This tendency aligns with H34’s generally more sensitive detection approach, which may prioritize completeness over strict precision. Aspect-wise, north-, northeast-, and southeast-facing slopes—which exhibit diverse snow retention and melting dynamics—tend to show higher rates of both misses and false alarms in H34, particularly where topographic shading or variable solar input can complicate snow identification. In contrast, H43 maintains consistently low percentages of both misses and false alarms across these directional classes, suggesting a more robust and stable snow classification under varying topographic and illumination conditions.

In the ≥2,500 m elevation range—the highest and most snow-dominated terrain of the Alps—the H34 and H43 snow cover products display distinct behaviors in terms of classification performance, particularly regarding misses and false alarms. H34 exhibits a higher rate of misses (~1.16%) compared to H43 (~0.60%), suggesting that H34 applies a more selective snow classification approach under conditions of spectral and topographic complexity. Meanwhile, H43 reports a slightly higher false alarm rate (~0.08%) than H34 (~0.03%). When aspect is considered, north-, northeast-, and northwest-facing slopes, which typically favor longer snow retention, show relatively high miss rates in H34 compared to H43. This suggests that H34 may underrepresent snow extent in shaded or cold-facing slopes. Likewise, east- and southeast-facing aspects, where snowmelt tends to occur earlier due to increased solar exposure, also exhibit higher misses in H34, possibly due to greater variability in snow cover or mixed-pixel effects. H43, in contrast, maintains a more stable distribution of both misses and false alarms across aspect classes, indicating consistent snow detection performance despite the ruggedness of the terrain.

**Figure 12:**
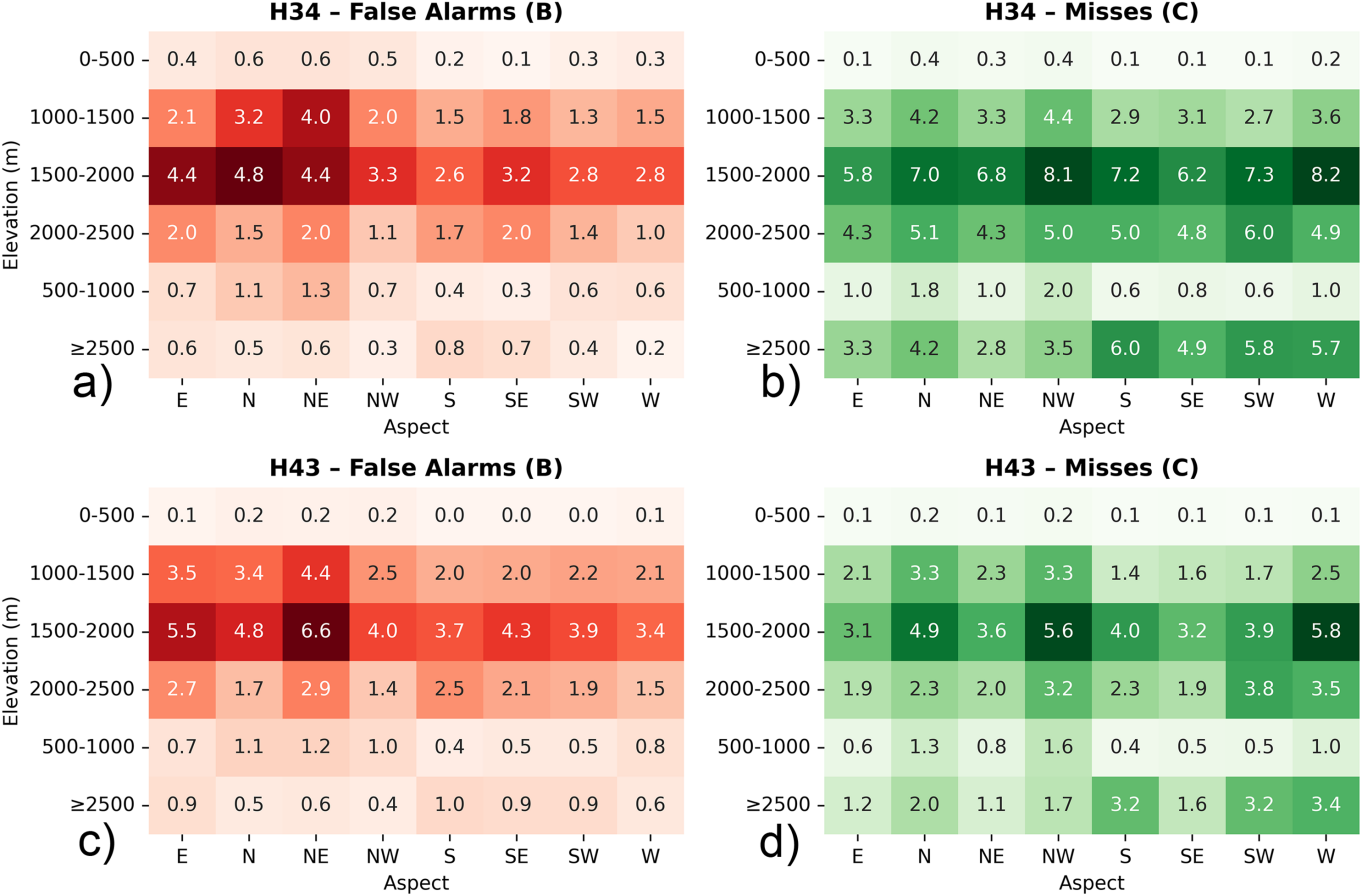
Heatmap diagrams of: (A) H34 false alarms, (B) H34 misses, (C) H43 false alarms, and (D) H43 misses, by elevation and aspect for Turkey. Metrics are expressed as percentages to ensure consistency between H34 and H43 products with differing spatial resolutions.

In the 1,000–1,500 m elevation range in Turkey, H34 and H43 snow products show differences in how they identify and miss snow-covered areas (cf. Fig. 12). Based on normalized values, H34 has a lower false alarm rate (~0.23%) than H43 (~0.29%), meaning it is slightly more cautious when classifying snow presence. This may help reduce overestimation of snow in areas where snow is thin or transient. On the other hand, H34 reports a higher rate of misses (~0.36%) compared to H43 (~0.23%), which shows that H34 may not capture some snow-covered areas in this elevation zone. These could be areas with patchy snow, terrain shadowing, or mixed surfaces, where snow detection is more difficult. When aspect is considered, north- and northeast-facing slopes—which typically hold snow longer—tend to show more misses in H34, while east-facing slopes contribute to slightly higher false alarms in H43. These differences reflect how each product responds to topographic and radiometric conditions that affect snow visibility.

In the 1,500–2,000 m elevation range in Turkey, H34 and H43 snow products show noticeable differences in both false alarm and miss rates. H34 has a lower false alarm rate (~0.39%) compared to H43 (~0.50%), meaning H34 is less likely to label snow-free areas as snow-covered. This may help reduce overestimation in areas where terrain or lighting conditions could cause confusion. At the same time, H34 has a higher miss rate (~0.79%) than H43 (~0.48%), suggesting that it may not detect snow in some locations where H43 does. These misses in H34 could be linked to shaded areas, early melt zones, or areas with thin or patchy snow cover. Regarding aspect, north- and east-facing slopes tend to show more misses in H34, possibly because of their longer snow retention and more challenging radiometric conditions. H43, while more inclusive in snow detection, tends to produce slightly more false alarms, especially on slopes where surface brightness varies.

In the 2,000–2,500 m elevation range, H34 and H43 show different behaviors in terms of snow classification. H34 has a lower false alarm rate (~0.17%) than H43 (~0.23%), which means H34 is more selective in identifying snow and is less likely to wrongly label snow-free areas as snow-covered. However, H34 reports a higher miss rate (~0.55%) compared to H43 (~0.29%), suggesting that H34 may not fully capture snow presence in certain parts of this elevation zone. These misses can occur in areas with complex terrain, partial snow coverage, or shaded slopes, where snow is more difficult to detect. When aspect is considered, north- and northeast-facing slopes contribute to slightly higher miss rates in H34, while H43 provides more consistent detection across aspects, though with a small increase in false alarms. The overall trend indicates that H43 is more inclusive in snow detection, while H34 continues to prioritize avoiding over-detection.

At the highest elevation band (≥2,500 m), snow is expected to be widespread and persistent. In this zone, H34 maintains a low false alarm rate (~0.06%), slightly lower than H43 (~0.08%), indicating that it rarely labels snow-free areas as snow-covered. However, misses are more common in H34 (~0.50%), while H43 shows a lower miss rate (~0.24%), suggesting that H43 is more successful in capturing snow presence under high-elevation conditions. The higher omission in H34 may result from the effects of slope, shading, or spectral confusion with bright surfaces like rock or glacier edges. Aspect analysis shows that H34 tends to miss more snow on north- and northwest-facing slopes, which generally retain snow longer. H43 performs more evenly across directional slopes, with only a slight increase in false alarms, pointing to its consistent classification behavior even in complex topography.

**Figure 13:**
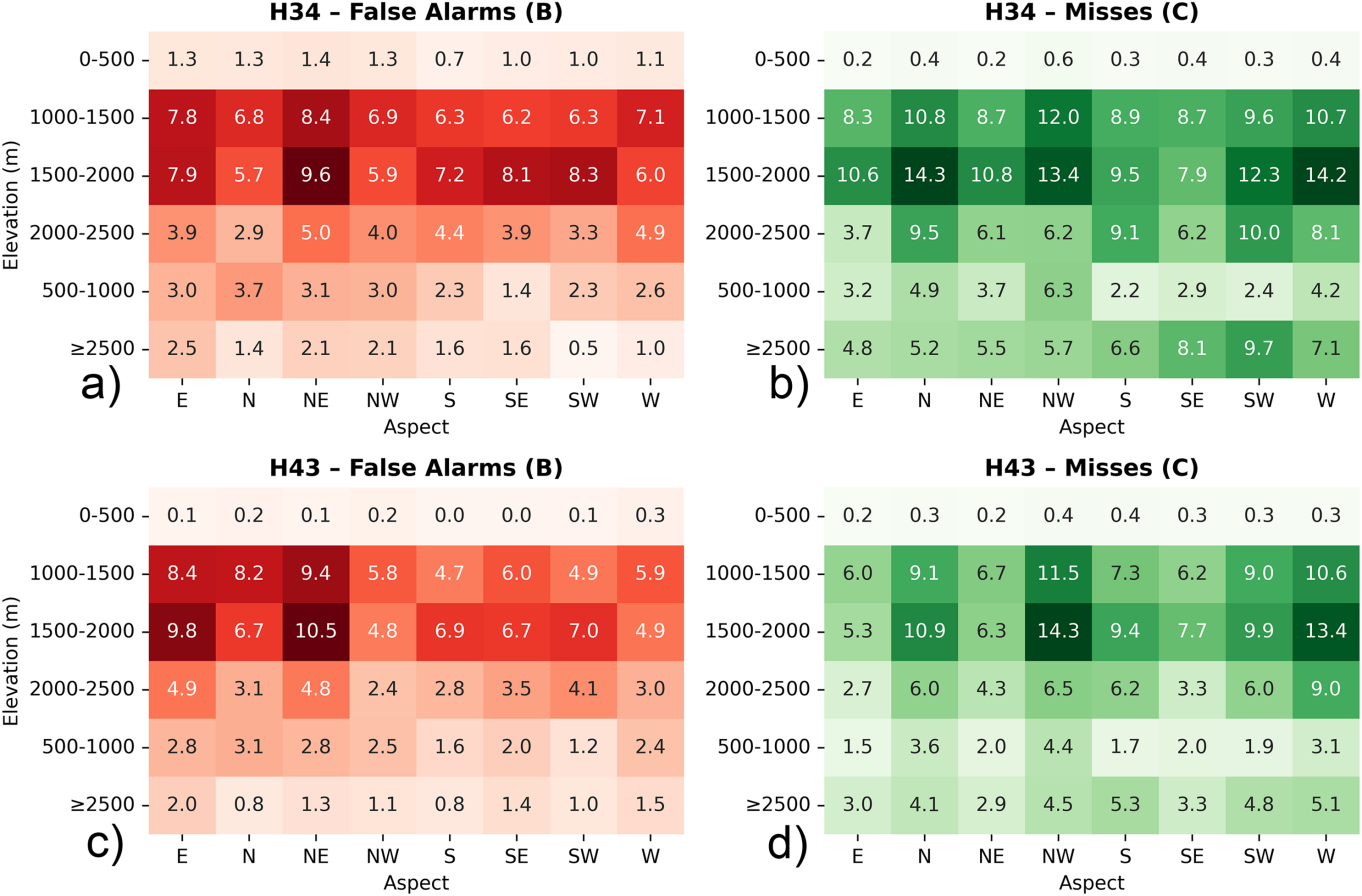
Heatmap diagrams of: (A) H34 false alarms, (B) H34 misses, (C) H43 false alarms, and (D) H43 misses, by elevation and aspect for Georgia-Caucasus. Metrics are expressed as percentages to ensure consistency between H34 and H43 products with differing spatial resolutions.

In Georgia, for the 1,000–1,500 m elevation range, both H34 and H43 products show relatively close values for false alarms and misses, though with some differences (cf. Fig. 13). H34 reports a slightly higher miss rate (~1.08%) than H43 (~0.92%), which means that H34 leaves out more snow-covered areas in this elevation zone. These misses could result from partial snow cover, terrain shadows, or radiometric complexity. False alarm rates between the two products are quite similar. H34 shows a false alarm rate of ~0.78%, while H43 is at ~0.77%, indicating that both products have a comparable tendency to overestimate snow presence in this elevation class. Aspect-based trends reveal that H34 has somewhat more frequent misses on north-facing and northeast-facing slopes, while H43 performs more evenly across aspect classes. Small variations in terrain exposure and snow retention may explain these differences.

In the 1,500–2,000 m elevation zone, both H34 and H43 show moderate levels of omission and commission errors, but with slight differences in magnitude. H34 has a miss rate of ~1.30%, while H43 is slightly lower at ~1.08%. This suggests that H43 detects more snow-covered areas in this elevation range, possibly due to a less restrictive detection threshold or better sensitivity to transitional snow conditions. For false alarms, the two products are again close in value. H34 records a rate of ~0.81%, while H43 is at ~0.79%. This indicates that both products show similar levels of over-detection in areas where snow may be patchy, melting, or confused with other bright surfaces. When examining aspects, north-facing and east-facing slopes contribute to the differences in omission rates, especially in H34. In these directions, snow tends to persist longer, which can challenge detection under certain lighting and viewing conditions.

Within 2,000–2,500 m, snow cover is more consistent, but detection performance still varies between the products. H34 shows a higher miss rate (~0.81%) compared to H43 (~0.61%), meaning H43 captures a slightly larger portion of the snow-covered area in this elevation band. This can be helpful in ensuring completeness in mountainous snow monitoring. False alarms are also somewhat different. H34 records a rate of ~0.45%, while H43 is slightly lower at ~0.40%, indicating both products are relatively stable in avoiding over-detection, with a small advantage for H43. In terms of terrain orientation, north- and northeast-facing slopes again show a larger share of H34’s misses.

And finally, in the highest elevation class (≥2,500 m), H34 shows a higher miss rate (~0.72%) than H43 (~0.46%), meaning it is more likely to leave out snow-covered areas in this range. This could be due to mixed terrain conditions or the presence of persistent snow that is harder to separate from other bright surfaces. False alarm rates are low for both products, but H34 reports a slightly higher value (~0.18%) than H43 (~0.14%). Both products perform well in avoiding over-detection at these altitudes, where snow presence is typically stable. Aspect-wise, the pattern continues: H34 tends to miss more snow on cold-facing slopes, especially in complex terrain, while H43 maintains more balanced performance across aspect classes.

### Land cover-based performance assessment

**Figure 14:**
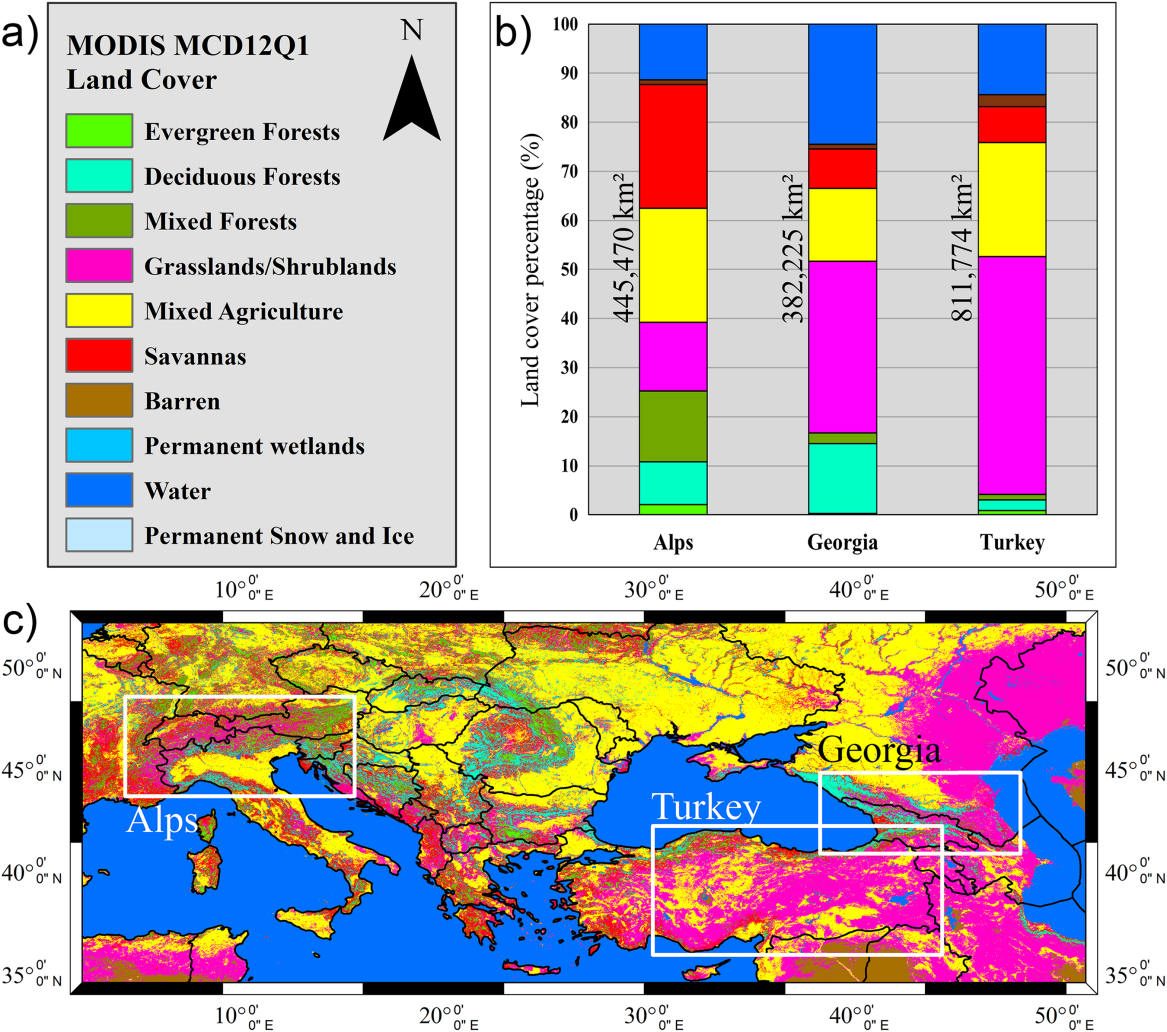
(A) Land cover classes from the MODIS MCD12Q1 product after merging, (B) corresponding area percentages, and (C) land cover composition across the three validation regions.

Figure 14 presents the land cover composition across the three validation regions, derived from the MODIS MCD12Q1 Land Cover Type product at 500 m spatial resolution, developed under the International Geosphere-Biosphere Programme (IGBP) (Friedl et al., 2010; Sulla-Menashe & Friedl, 2022). This global product provides standardized land cover classification using 17 original IGBP classes. However, for the purpose of this study, similar land cover types were merged, resulting in a simplified 10-class scheme (cf. Table 4) to improve interpretability while retaining essential surface characteristics relevant to snow detection, as also applied in Dobreva & Klein (2011), Kuter, Akyurek & Weber (2018), and Kuter (2021).

**Figure 15:**
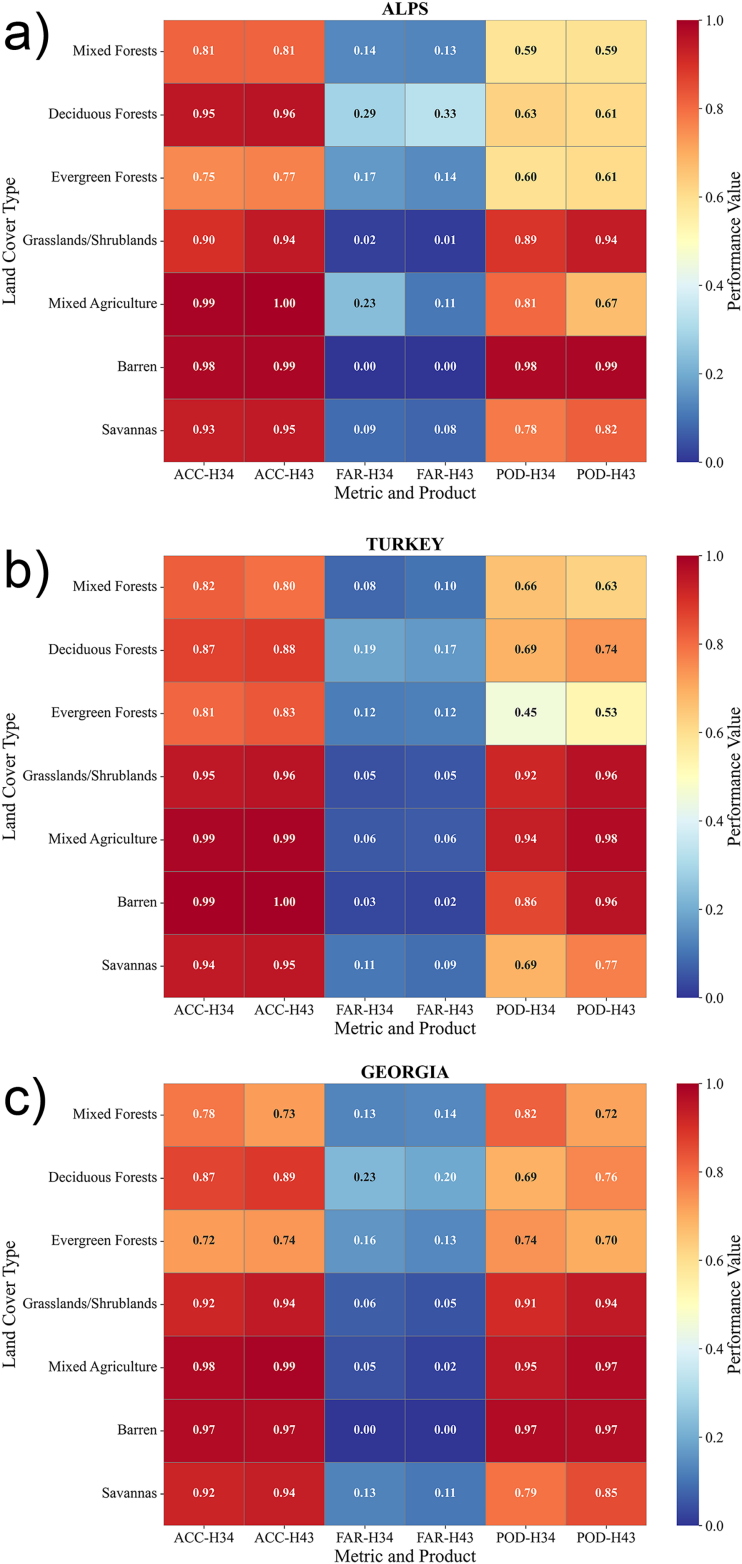
Heatmaps of performance metrics for the H34 and H43 products with respect to land cover over (A) the Alps, (B) Turkey, and (C) the Georgia–Caucasus region.

Across all validation regions, the H43 product consistently exhibited superior performance compared to its predecessor H34, showing higher POD and overall ACC, while maintaining comparable or slightly reduced FAR values. The impact of land cover heterogeneity was particularly evident in forested and mixed vegetation zones, where snow classification is more complex due to canopy obstruction, varying spectral reflectance, and sub-pixel snow variability. The stratified evaluation based on land cover types, illustrated in Fig. 15, underscores how the detection performance of each product is influenced by surface conditions, providing a more detailed assessment of their reliability across diverse land cover environments.

In the European Alps, land cover types such as Evergreen Forest and Mixed Forest were associated with lower POD values for both H34 and H43 products, primarily due to canopy-induced snow misclassification. Dense forest cover obscures the underlying snow signal, leading to detection challenges. In contrast, open areas like grasslands and shrublands showed significantly higher POD values, reflecting improved snow retrieval where vegetation interference is minimal. H43 demonstrated reduced false alarm rates in forested zones compared to H34, resulting in higher classification accuracy.

Similar patterns were observed in the Turkey region. Land cover types such as grasslands, barren areas, and agricultural lands exhibited strong detection performance, with high POD and ACC values. Forested zones, particularly deciduous and mixed forests, presented lower detection rates, again highlighting the limitations of VIS/IR snow mapping in densely vegetated environments. Agricultural areas showed some increase in misclassification errors, likely due to spectral confusion stemming from seasonal land-use dynamics and mixed-pixel effects. Despite this, H43 consistently outperformed H34, providing higher accuracy across all land cover categories, even in complex terrain.

In the Georgia-Caucasus region, the presence of mixed vegetation, croplands, and forested landscapes contributed to increased FAR values, illustrating the difficulty of discriminating snow from vegetated surfaces under heterogeneous conditions. Nevertheless, H43 achieved improved POD and reduced false detections in these challenging classes, outperforming H34 in both detection capability and classification stability. Open land covers, particularly grasslands and barren lands, delivered the most accurate snow detection results, underscoring the importance of surface clarity for VIS/IR-based snow algorithms.

Overall, both products exhibit strong agreement in terms of POD, FAR, and ACC, with regional and land cover–specific variations remaining within acceptable margins. H43 generally achieves slightly higher POD and ACC values in most land cover types, particularly in grasslands/shrublands and savannas, where detection capability and classification accuracy are notably high. However, these improvements are not uniform across all regions or classes. In some cases—such as POD in mixed forests or agriculture in the Alps—H34 records higher or comparable values, while FAR values remain closely aligned or slightly lower in H43. These subtle differences reflect expected variability due to regional snow characteristics and land surface heterogeneity. Importantly, no systematic or consistent superiority is observed between the two products across all metrics and land cover types. The findings suggest that both H34 and H43 provide reliable snow cover detection performance under a range of surface conditions. Given the consistency of their behavior, H43 may be considered a robust continuation of the H34 product line, supporting its applicability in operational snow monitoring and climate-related applications over heterogeneous landscapes.

## Conclusions

This study presents the first large-scale evaluation of the EUMETSAT H SAF H43 snow cover product, derived from the Meteosat Third Generation Flexible Combined Imager (MTG-FCI). Using both MODIS-based reference snow maps and *in-situ* WMO synoptic snow-depth observations, H43 performance was assessed across diverse geographical settings, land-cover types, elevation bands, and slope aspects.

The results indicate that H43 exhibits modest yet consistent improvements over its predecessor H34 across key accuracy metrics, showing slightly higher probability of detection and overall accuracy while maintaining false alarm ratios below 30%. These gains, although numerically limited in some regions (0.004–0.01 in POD), are systematic across different terrain types, suggesting improved detection stability and reliability. The benefits are most noticeable in mountainous areas, where miss rates decreased by up to 40% above 2,000 m elevation. Validation using *in-situ* observations supports these trends, though the statistical robustness in the Georgia–Caucasus region remains constrained by the limited number of ground stations. Cross-validation with MODIS-based snow maps provides a spatially comprehensive confirmation of these results.

When interpreting the validation outcomes, it is essential to recognize the inherent biases and limitations of MODIS-derived reference data, which influence the apparent performance of H34 and H43. The MODIS MOD10A1 product, while well established for global snow mapping, is susceptible to cloud contamination, forest-canopy effects, and illumination-related uncertainties in complex terrain. These factors can lead to spatially inconsistent reference conditions, particularly in mountainous and forested regions where snow cover varies rapidly within a single 500 m pixel. Consequently, part of the observed discrepancies between MODIS and the H SAF products may stem from reference data limitations rather than true product errors.

A central motivation of this work is to highlight the added value of geostationary optical observations for operational snow monitoring. Compared with polar-orbiting systems such as MODIS and VIIRS, the MTG-FCI sensor acquires images every 10 min, theoretically yielding up to 144 scenes per day, of which approximately 65–70 daytime acquisitions are used to generate the H43 snow product under solar illumination. This high temporal frequency enables near-continuous monitoring and substantially reduces cloud-related data loss. In this study, cloud-induced misses (cases where a station reported snow but the satellite pixel was cloud-covered) accounted for 28% (H34) and 30% (H43) in the Alps and 25% (H34) and 26% (H43) in Turkey, whereas MODIS missed about 47–49% under similar conditions. These results demonstrate that Meteosat observations reduce cloud-related data loss by nearly half, consistent with previous findings showing that merging 32 SEVIRI scenes per day reduces cloud coverage by approximately 37% compared to MODIS daily composites (Sürer & Akyürek, 2012; Sürer, Parajka & Akyürek, 2014).

The innovation of this study lies in establishing a baseline, multi-source validation framework for the first MTG-based H SAF snow product. The findings demonstrate that H43 provides incremental yet meaningful improvements in accuracy and reliability while ensuring operational continuity within the H SAF framework. As part of the long-standing Meteosat mission series, H43 complements polar-orbiting products and reinforces Europe’s geostationary capability for continuous, near-real-time cryospheric monitoring in a changing climate.

## Appendix–H43 algorithm flowchart

The overall processing scheme of the H43 product is illustrated in the flowchart provided in this appendix. The diagram summarizes the main algorithmic steps, including input data acquisition, cloud and snow discrimination, and generation of the final snow cover extend output. Each processing component reflects the operational logic implemented within the H SAF framework to ensure consistency, reliability, and traceability across the product chain. The flowchart serves as a visual complement to the methodological descriptions presented in the main text, offering a concise overview of the sequential workflow applied in the H43 algorithm.

## Additional Information and Declarations

### Data Availability

The following information was supplied regarding data availability:

The dataset is available at Zenodo:

Kuter, S. (2025). Updated Validation data for H43 snow cover product from MTG-FCI [Data set]. Zenodo. https://doi.org/10.5281/zenodo.16792205.
